# Potential risk of *Batrachochytrium salamandrivorans* in Mexico

**DOI:** 10.1371/journal.pone.0211960

**Published:** 2019-02-12

**Authors:** M. Delia Basanta, Eria A. Rebollar, Gabriela Parra-Olea

**Affiliations:** 1 Departamento de Zoología, Instituto de Biología, Universidad Nacional Autónoma de México, AP, Tercer Circuito Exterior s/ n, Ciudad Universitaria, Ciudad de México, México; 2 Posgrado en Ciencias Biológicas, Unidad de Posgrado, Edificio A, 1° Piso, Circuito de Posgrados, Ciudad Universitaria, Coyoacán, C.P., Ciudad de México, México; 3 Centro de Ciencias Genómicas, Universidad Nacional Autónoma de México, Cuernavaca, Morelos, México; Vanderbilt University School of Medicine, UNITED STATES

## Abstract

The recent decline in populations of European salamanders caused by the chytrid fungus *Batrachochytrium salamandrivorans* (*Bsal*) has generated worldwide concern, as it is a major threat to amphibians. Evaluation of the areas most suitable for the establishment of *Bsal* combined with analysis of the distribution of salamander species could be used to generate and implement biosecurity measures and protect biodiversity at sites with high salamander diversity. In this study, we identified the areas most suitable for the establishment of *Bsal* in Mexico. Mexico has the second-highest salamander species diversity in the world; thus, we identified areas moderately to highly suitable for the establishment of *Bsal* with high salamander diversity as potential hotspots for surveillance. Central and Southern Mexico were identified as high-risk zones, with 13 hotspots where 30% of Mexican salamander species occur, including range-restricted species and endangered species. We propose that these hotspots should be thoroughly monitored for the presence of *Bsal* to prevent the spread of the pathogen if it is introduced to the country.

## Introduction

Chytridiomycosis, an emergent disease caused by the fungal pathogen *Batrachochytrium dendrobatidis* (*Bd*), has caused alarming population collapses and extinctions of amphibians worldwide [1–7]. In many regions throughout the world, *Bd* infections have transitioned from epidemic to endemic states after declines occurred, and at present, some amphibian populations are either stable or recovering after more than a decade since the disease outbreaks occurred [4, 8, 9]. The recent emergence of a second chytrid fungus, *Batrachochytrium salamandrivorans* (*Bsal*), has unleashed great concern among researchers and conservation agencies since this pathogen has already caused die-offs of native salamander species in Europe [10]. The potential effects of *Bsal* infections in other regions of the world are still unclear. However, recent studies in European salamander populations determined that the combination of *Bsal´s* transmission strategy, virulence and host population dynamics could have catastrophic effects on naïve salamander populations [11, 12], including those species that were not previously affected by *Bd*. The urgency of this situation requires estimation of the potential effect of *Bsal* if introduced to naïve regions.

The available ecological data can provide some indicators regarding the potential of *Bsal* to contribute to the global decline in amphibians. Although *Bd* affects all groups of amphibians, experimental studies have documented that *Bsal* infections are harmful to urodeles even at very low *Bsal* zoospore levels [11, 12], whereas anurans can coexist with *Bsal* infections and act as reservoirs [12, 13]. *Bsal* physiological studies have shown that the thermal growth range of *Bsal* in the laboratory is between 5°C and 25°C, with optimal growth between 10°C and 15°C [10], which is considerably lower than the optimal temperature range of *Bd* (between 17°C and 25°C) [14]. However, recent surveys in Vietnam and China found *Bsal* in ponds and streams with water temperatures between 20°C and 25°C [15, 16], suggesting a wide thermal niche for this pathogen.

Since *Bsal* is lethal to some urodele species, its spread to naïve regions with high salamander diversity, such as North America, could cause significant reductions in amphibian diversity [11, 17, 18]. Thus, areas that are moderately to highly suitable for *Bsal* with high salamander diversity could be considered as hotspots in which surveillance strategies should be implemented to prevent potential amphibian declines.

The use of ecological niche modeling (ENM) to infer the suitable distribution of *Bsal* could provide an estimate of sites with the potential risk of infection based on bioclimatic variables and salamander distributions [18–23]. Yap et al. [18] created a species distribution model for *Bsal* using the native ranges of the three putative native *Bsal* host species in Asia (*Cynops cyanurus*, *C*. *pyrrhogaster*, and *Paramesotriton deloustali*) and projected these results to North America. These analyses identified the southern part of the Appalachian Mountains, the Pacific Northwest, the Sierra Nevada, and the mid-Atlantic as high-risk zones in the USA and the Sierra Madre Oriental (SMO) and the Trans-Mexican Volcanic Belt (TVB) as high-risk zones in Mexico. However, Yap et al. [18] analyzed the native host niche under the assumption that it is a proxy for the *Bsal* ecological niche.

Since *Bsal* has been found in different environmental conditions outside the native host range of Asia (introduced areas in Europe), we constructed a potential distribution model to identify areas susceptible to invasion. We modelled *Bsal*’s niche considering environmental layers and the occurrences of *Bsal* in its native and invasive areas to create projections of *Bsal* suitability in Mexico. Based on the obtained *Bsal* model and the salamander richness distribution, we identified major hotspots for salamander decline in the event of *Bsal* introduction in Mexico. This information will be relevant to implement conservation strategies in Mexico, which has the world’s second-highest salamander richness, with 146 described species [24].

## Materials and methods

### *Batrachochytrium salamandrivorans* potential distribution model

To assess the potential distribution of *Bsal* in Mexico, we mapped its climatic niche based on environmental layers and *Bsal* occurrences from both Europe and Asia [25] (S1 Fig). *Bsal* occurrence records were obtained from Martel et al. [11], Laking et al. [15], Yuan et al. [16], Spitzen-van der Sluijs et al. [26], and Beukema et al. [27] (S1 Table).

The model was built using the maximum entropy algorithm MaxEnt [23]. This software estimates the probability of species occurrence by finding the distribution of maximum entropy, which is subject to constraints defined by the environmental variables being analyzed [23]. To avoid model overfitting and multicollinearity of predictors [28], we selected the non-correlated variables chosen by MaxEnt. Briefly, we first ran MaxEnt using all 19 bioclimate layers from Wordclim [29] at 30 arcsecond (~1 km) resolution to let the software select the variables (S2 Table). Then, we calculated pairwise Pearson correlations between the variables using ENM tools [30], and we selected those with the maximum contribution percent in the model and with a Pearson’s *r* < 0.75 (S3 Table): mean diurnal range (Bio2), maximum temperature of warmest month (Bio5), temperature annual range (Bio7), precipitation seasonality (Bio15), precipitation of warmest quarter (Bio18), and precipitation of coldest quarter (Bio19).

The MaxEnt model was optimized using the ENMeval package [31] implemented in R 3.2.4 [32], which provides an automated method to execute MaxEnt models across a user-specified range of regularization multiplier (RM) values and feature combinations (FCs). We set the RM range from 0.5 to 2.5 with increments of 0.5 and three FCs, i.e., linear (L), linear and quadratic (LQ), and linear, quadratic and product (LQP), resulting in 15 possible combinations of features and regularization multipliers. The fine-tuned MaxEnt models were made by seeking the lowest delta value of Akaike’s information criterion corrected for small samples sizes (AICc) among candidate models, which reflects both model goodness-of-fit and complexity providing the most conservative results. In addition, AICc balances predictability against model complexity due to penalties for overparameterization [21, 31]. The models were built based on an approach proposed by Phillips [33]. Briefly, we modelled *Bsal*’s niche using the native area (Asia) to train the model (occurrences and background) and the invasive area (Europe) as testing data. We also used a block method to generate AUC scores [31].

We selected the model with the lowest delta AICc score, which had a parametrization of regularization multiplier of 2.5 and a LQP feature combination; it exhibited good predictive power, with high accuracy and an average test AUC value of 0.87 (S4 Table, S2 Fig). This model was used to project *Bsal* in Mexico to create the bioclimatic suitability model (logistic output). Because the logistic output from Maxent ranges from 0 to 1, with 0 indicating unsuitable habitat and 1 indicating the highest suitability, we reclassified the predicted values using 0.25 intervals to obtain four suitability classes: no suitability when values were less than 0.25, low suitability when the occurrence probability ranged between 0.25 and 0.5, moderate suitability when the values ranged between 0.5 and 0.75, and high suitability when the values were greater than 0.75 [34–36]. In addition, we defined the potential presence and absence areas in Mexico using the minimum training presence threshold, which correspond to the lowest predicted presence value of an occurrence record [37].

### Salamander distribution and richness areas

We estimated salamander richness in Mexico by overlapping 161 distribution maps [18]. 136 of the distribution maps were obtained from the IUCN Red List [38], and the remaining 25 were expert-based maps (*Aquiloeurycea cafetalera*, *Bolitoglossa chinanteca*, *B*. *odonelli*, *Bradytriton silus*, *Chiropterotriton sp*. *I*, *Chiropterotriton sp*. *C*, *Chiropterotriton sp*. *E*, *Chiropterotriton sp*. *F*, *Chiropterotriton sp*. *G*, *Chiropterotriton sp*. *H*, *Chiropterotriton sp*. *J*, *Chiropterotriton sp*. *K*, *Chiropterotriton aureus*, *Chiropterotriton chico*, *Chiropterotriton cieloensis*, *Chiropterotriton infernalis*, *Chiropterotriton miquihuanus*, *Chiropterotriton nubilus*, *Isthmura corrugata*, *Isthmura sierraoccidentalis*, *Thorius hankeni*, *T*. *longicaudus*, *T*. *maxillabrochus*, *T*. *pinicola*, *T*. *tlaxiacus*). Expert-based maps were obtained based on records from the Global Biodiversity Information Facility (GBIF, http://www.gbif.org), National Biodiversity Information System of Mexico (SNIB) and published papers [39–41]. These records were carefully reviewed, and we added a 1-km buffer radius to each record according the registered plethodontids home ranges [42]. Moreover, we modified distributions of three species from IUCN (*Ambystoma granulosum*, *A*. *rivulare*, *Chiropterotriton multidentatus*) considering the occurrences and last updates published [43, 44]. We also consulted the International Union for Conservation of Nature (IUCN)-The Global Amphibian Assessment (GAA) to obtain the conservation status for all Mexican salamanders listed in the database. We used ArcGIS 10.2 [45] to produce all GIS layers and calculate the distribution area. In addition, we used the R statistical software package to overlap the species distribution and perform the richness map at a resolution of 30 arcseconds (~1 km^2^). Expert-based maps and modified maps are available at https://github.com/delibasanta/Mexican-salamanders.git

### Geographic overlap

We created a salamander-vulnerability model by calculating the overlap of suitable areas of *Bsal* and the salamander-richness distribution. This model retrieved biodiversity hotspots in which *Bsal* has suitable bioclimatic conditions (*Bsal* suitability >0.5) and salamander diversity is high (more than five salamander species).

## Results

### *Batrachochytrium salamandrivorans* has several potential suitable regions in Mexico

**Fig 1:**
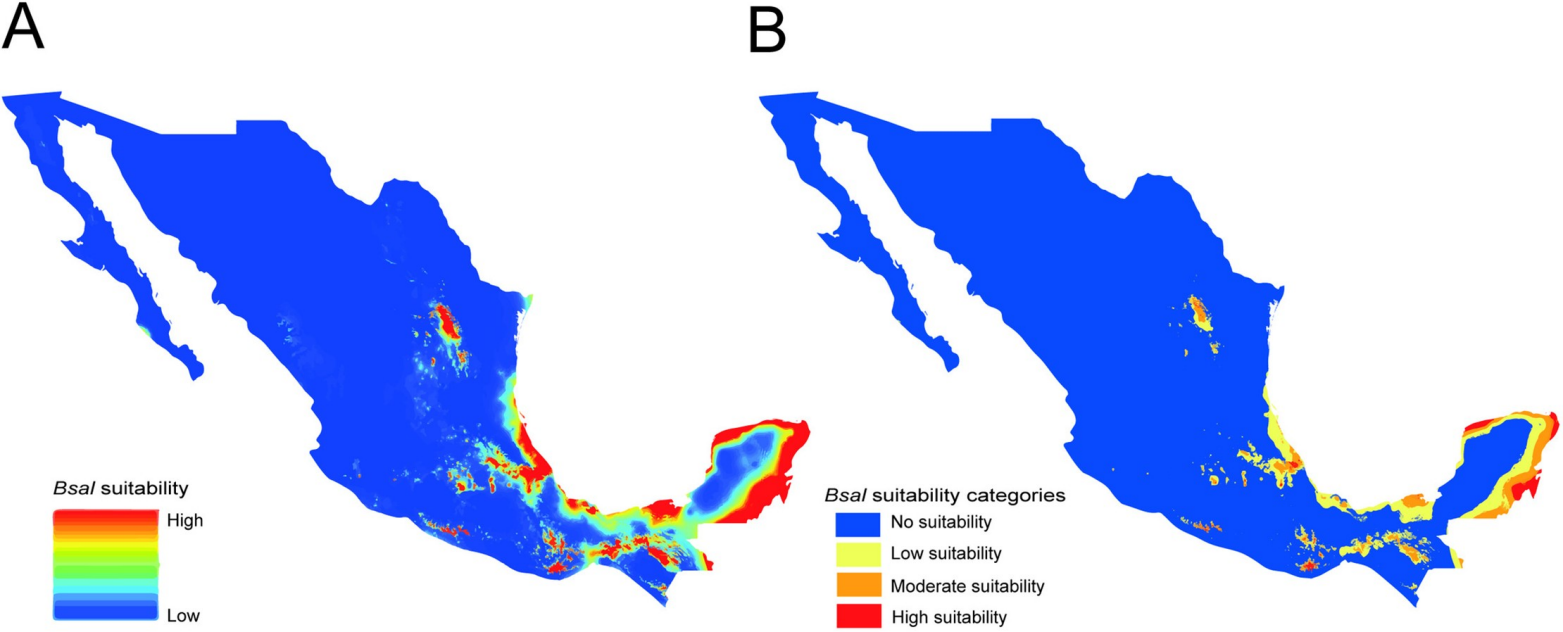
Suitability model for *Bsal* in Mexico. (A) Continuous model (B) Categorized model.

We found that areas from the Sierra Madre Oriental (SMO), Trans-Mexican Volcanic Belt (TVB), Sierra Madre del Sur (SMS), Mexican Gulf and Yucatan Peninsula were the most suitable areas for *Bsal* (Fig 1, S3 Fig). Of the six environmental variables, temperature annual range (Bio7), minimum temperature of the coldest month (Bio5) and precipitation seasonality (Bio15) had the largest contributions to the distribution model for *Bsal* (S5 Table). These three factors explained 91.3% of the modeled distribution. The contributions of the other factors, i.e., mean diurnal range (Bio2), precipitation of the warmest quarter (Bio18), and precipitation of the coldest quarter (Bio19) were 5.8%, 1.92%, and 0.97%, respectively. These results indicate that thermal conditions and precipitation seasonality were the most important variables of the obtained *Bsal* niche model.

### Salamander distribution in Mexico is highly heterogeneous

**Fig 2:**
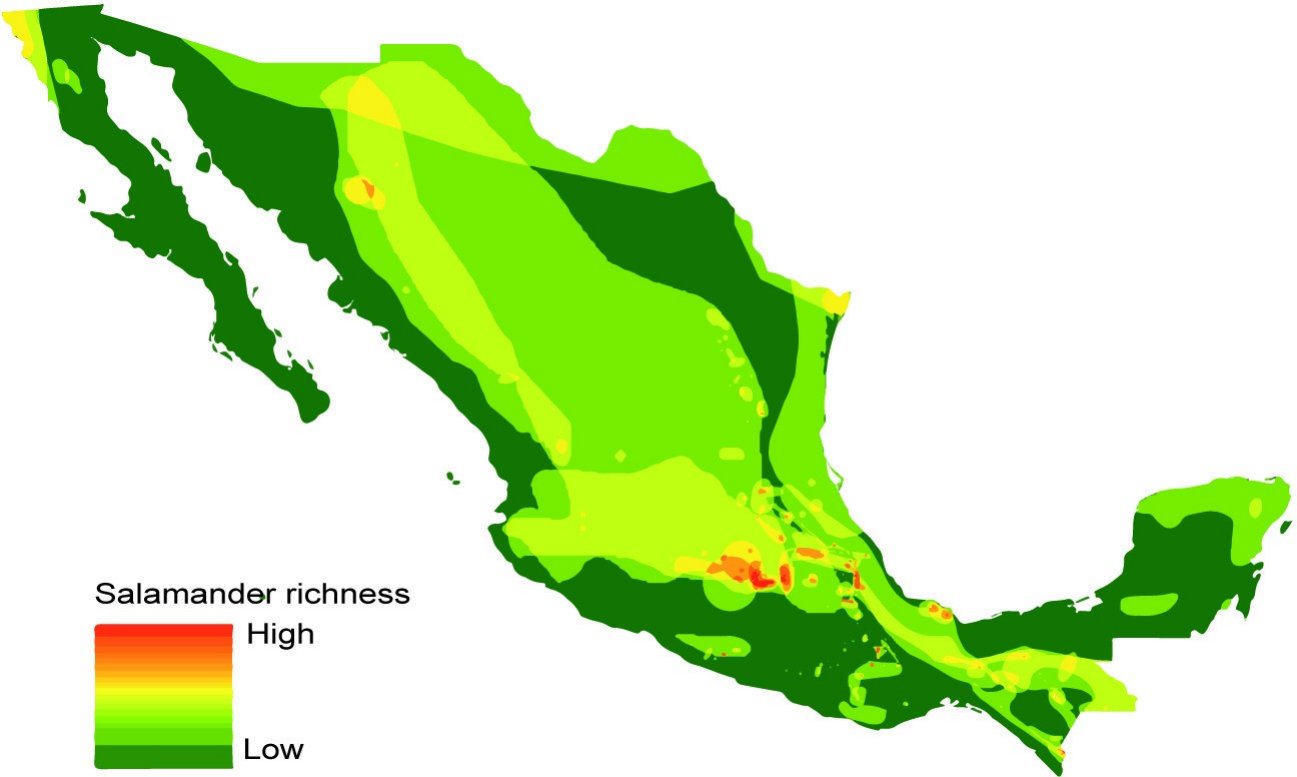
Map of salamander richness distribution in Mexico.

We obtained distribution maps for 161 salamander species (153 described species and 8 undescribed species, *i*.*e*., *Chiropterotriton* spp.) (S6 Table). These maps were used to generate a salamander richness map (Fig 2). The salamander distribution in the country is heterogeneous, with most of the species occurring in Central and Southern Mexico (Fig 2).

According to the areas in which salamanders occur, 44% of the salamander species exhibit a small distribution (<100 km^2^) and are endemic to Mexico (S6 Table). Moreover, most Mexican salamanders are threatened according the IUCN Red List classification, with 33% of the species listed as critically endangered, 29% as endangered, 8% as vulnerable, 5% as near threatened, 10% as least concern, 4% as data deficient, and 11% not evaluated (S6 Table).

Species from the family Plethodontidae represented 87.4% of the total number of species in the country, whereas Ambystomatidae, Salamandridae and Sirenidae represented 10.7%, 0.63% and 1.26%, respectively.

### Geographic overlap: High correspondence between *Bsal* suitability and salamander richness

**Fig 3:**
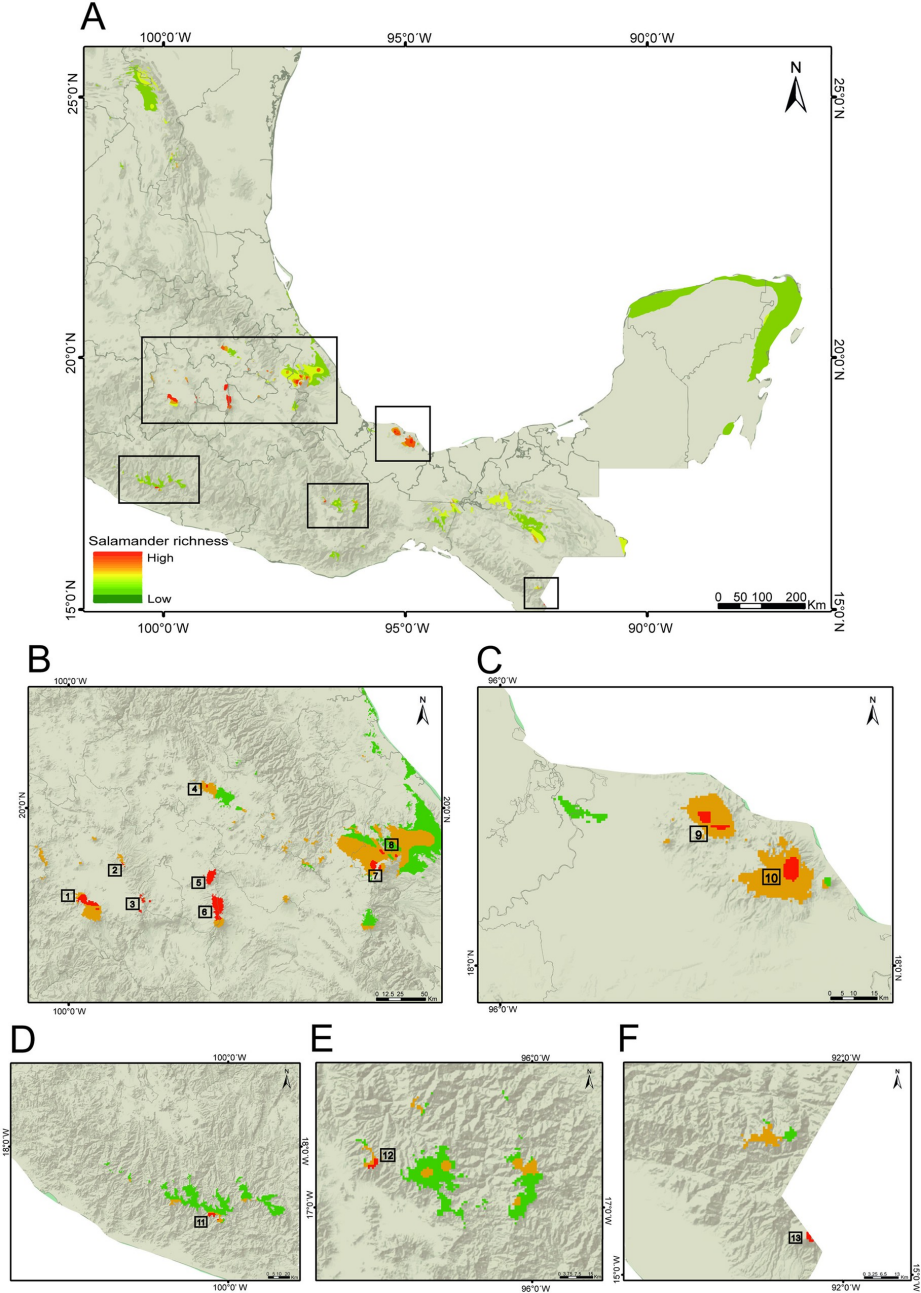
Overlap of salamander richness with moderate suitable and high suitable areas for *Bsal*. (A) Areas with hotspots. Hotspots identification in: (B) TVB. (C) Los Tuxtlas. (D) SMS in Guerrero. (E) Northern Oaxaca. (F) SMS in Chiapas.

**Fig 4:**
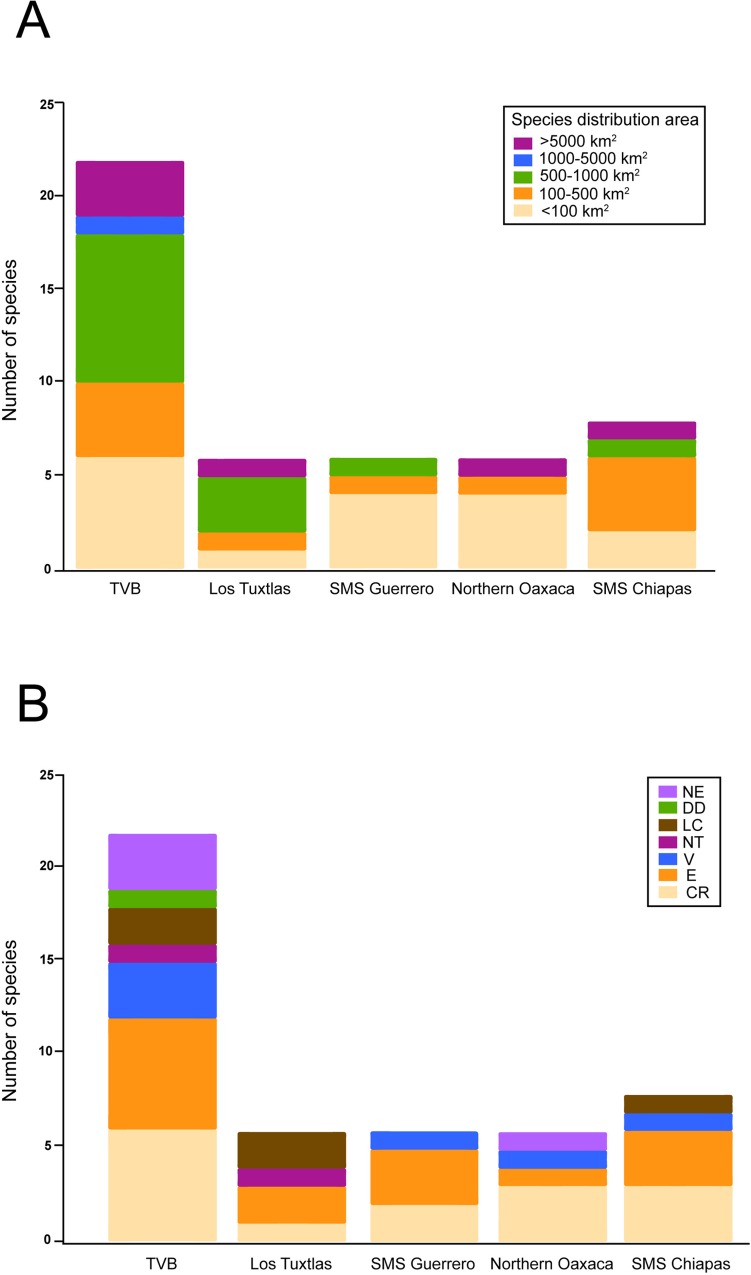
Relationship between hotspots and salamander species. (A) Number of salamander species present in the hotspot areas and their associated distribution range. (B) Number of salamander species present in the hotspot areas and the associated IUCN categories.

We found that 51% of salamander species of Mexico are present in areas that are suitable for *Bsal* (S4 Fig, S6 Table). We identified 13 hotspots as those suitable areas categorized as moderate and high suitability for *Bsal* (Fig 1B) in which five or more salamander species were present (Fig 3). All hotspots are located in Central and Southern Mexico: eight are located on the Trans-Mexican Volcanic Belt (TVB), two in Los Tuxtlas Veracruz, one in the Sierra Madre del Sur (SMS) in Guerrero, one in Northern Oaxaca, and one in SMS in Chiapas (Fig 3). These hotspots included 47 salamander species across seven genera, including species with a restricted geographical range (<100 km^2^) (Fig 4A, S6 and S7 Tables) and endangered species (Fig 4B, S6 Table).

## Discussion

Due to the potential risk of introducing *Bsal* to native regions through wildlife trade [13, 46], we used ENM and salamander distribution data to determine the potential areas and species that are most likely to be at risk of pathogen exposure should an introduction occur in Mexico. Areas with high salamander diversity have climatic conditions that appear to be suitable for the establishment of *Bsal* should an introduction occur. Considering the latter, the risk of *Bsal* arrival is critically important, and it is essential to monitor these areas where species loss would be considerable.

Our niche model predictions differ considerably from those obtained by Yap et al. [18], in which the authors estimated the habitat suitability for invasive *Bsal* in North America. Using Maxent and amphibian host occurrence records, Yap et al. [18] predicted areas of Sierra Madre Occidental (SMOc), Trans-Mexican Volcanic Belt (TVB), Sierra Madre del Sur (SMS), Baja California and Oaxaca as suitable for *Bsal* in Mexico. In contrast, our study predicted some parts of the TVB, SMS, Sierra Madre Oriental (SMO), Northern Oaxaca, Mexican Gulf and Yucatan Peninsula as suitable areas for *Bsal*. The discrepancies between Yap et al. [18] and our study (i.e., Baja California, SMOcc, Mexican Gulf and Yucatan Peninsula) can be explained by methodological differences in calibration areas and the occurrences used to estimate the potential range of *Bsal* in North America. Specifically, our study used the native niche of *Bsal* rather than the native Asian host distribution used by Yap et al. [18]. We consider that the use of *Bsal* ranges instead of *Bsal* host ranges will lead to more accurate results when modeling the potential invasive range of the pathogen.

The model obtained in this study predicted that the areas suitable for *Bsal* are mainly located in Central and Southern Mexico, including diverse environments such as tropical forests, pine forests and cloud forests. We found a high overlap between salamander richness and moderately to highly suitable areas for *Bsal*, which is reflected in the 13 identified hotspots. These hotspots are located in the most diverse regions with respect to amphibian species (including salamanders): east of the Trans-Mexican Volcanic Belt, Northern Oaxaca and Sierra Madre del Sur in Chiapas [47–49]. Thus, the arrival of *Bsal* in these areas will likely have an impact on amphibian communities that include non-susceptible species (e.g., anuran species) that could act as carriers and transmission vectors [13], in addition to highly susceptible species (e.g., salamander species) [12]. However, *Bsal* could have different strains with genetic physiological and virulence differences such as the case with *Bd* [50]. In terms of *Bsal*, Sabino-Pinto et al. [51] has already suggested the existence of more than one *Bsal* strains with differences in virulence. In this context, further investigations are needed to describe the genetic differences between *Bsal* strains and it’s effect on Mexican taxa to apply better conservation strategies.

Most Mexican salamander species have a restricted distribution, including those species that inhabit hotspots. Salamander communities mainly inhabit pine-oak forests, tropical forests and cloud forests [52]. These environments in Mexico are highly affected by anthropogenic activities, including deforestation and land use transformation [53–55]. Thus, habitat loss has been one of the main causes of species decline: major amphibian declines were observed between 1970 and 1980 east of the Trans-Mexican Volcanic Belt, Northern Oaxaca and Sierra Madre del Sur in Chiapas, which coincide with four of the hotspots identified in this study (hotspots 4, 11, 12 and 13). Lips et al. [56] reported population declines and local extinctions in the Pacific slope Sierra Madre del Sur in Guerrero (hotspot 11), Northern Oaxaca (hotspot 12) and Sierra Madre del Sur in Chiapas (hotspot 13). Later, Rovito et al. [57] documented declines in salamander populations of El Chico in Hidalgo (hotspot 4) and Cerro San Felipe in Oaxaca (hotspot 12), where populations of *Chiropterotriton* and *Pseudoeurycea*, respectively, were the most affected.

For Mexican amphibians, in addition to habitat loss, the presence of *Bd* has also been considered a threat. This pathogen was detected in individuals collected in the 1970s, suggesting that chytridiomycosis has affected amphibian populations since then [7, 56]. The presence of *Bd* has been corroborated in many localities, including all 13 hotspots identified in this study [58, 59]. The combined effects of *Bd* and *Bsal* together in amphibian populations are unknown, but we can only assume that they could dramatically affect the amphibian populations that are already threatened by habitat loss.

Conservation efforts for amphibians in Mexico should focus on preventing the arrival of *Bsal* and its transmission among populations. Amphibian trade restrictions are being implemented in the USA, Canada and the European Union, and Mexico should not be the exception. As the country with the second-highest salamander species diversity, Mexico is potentially at risk of facing dramatic declines upon the arrival of an emerging pathogen such as *Bsal*. If *Bsal* is detected in Mexico, immediate management actions to prevent its spread, such as restricting site-level access, especially in hotspots, should be considered.

## Conclusions

This study integrated ecological niche modeling of *Bsal* and salamander distribution in Mexico and found high overlap between them. The areas most suitable for *Bsal* in Mexico are Central and Southern Mexico, which coincide with the highest salamander richness areas and with the largest number of endemic and threatened species. We identified 13 areas as potential hotspots for population risk with both high salamander diversity and areas that are moderately to highly suitable for *Bsal*. We propose that the hotspots should be monitored for the presence of *Bsal* to prevent the spread of the pathogen if it is introduced to Mexico.

## Supporting information

S1 Fig Occurrences and areas of *Batrachochytrium salamandrivorans* (*Bsal*) used to build the model. (TIF)

S2 Fig AICc and AUC values of *Bsal* models obtained with ENMeval. (TIF)

S3 Fig Binary model for *Bsal* obtained with the minimum training presence threshold. Areas of potential presence are in black, and areas of potential absence are in gray.
(TIF)

S4 Fig Number of salamander species grouped by genus that are present or absent in *Bsal*-suitable areas. (TIF)

S1 Table Occurrence data used for *Bsal* ecological niche model. (DOCX)

S2 Table Contribution of 19 bioclimate layers from Wordclim that made greatest contribution to the model constructed with MaxEnt. (DOCX)

S3 Table Pairwise Pearson correlations of 19 bioclimatic variables. Variables selected with less than *r* = 0.75 are in bold.
(DOCX)

S4 Table ENMeval models results. (DOCX)

S5 Table Variable contributions to *Bsal* distribution model. (DOCX)

S6 Table Salamander species of Mexico, range, IUCN status, and relationship with *Bsal* model and hotspots. Hotspots = pixels with *Bsal* suitability values greater than 0.5 and more than five salamander species. IUCN status: critically endangered (CR), endangered (E), vulnerable (V), near threatened (NT), least concern (LC), data deficient (DD), and not evaluated (NE).
(DOCX)

S7 Table Species present on hotspots. (DOCX)
