# Supplementary figures and images for: Potential risk of Batrachochytrium salamandrivorans in Mexico
Source: PLoS One. 2019 Feb 12;14(2):e0211960. doi: 10.1371/journal.pone.0211960 (PMC6372179; doi:10.1371/journal.pone.0211960)

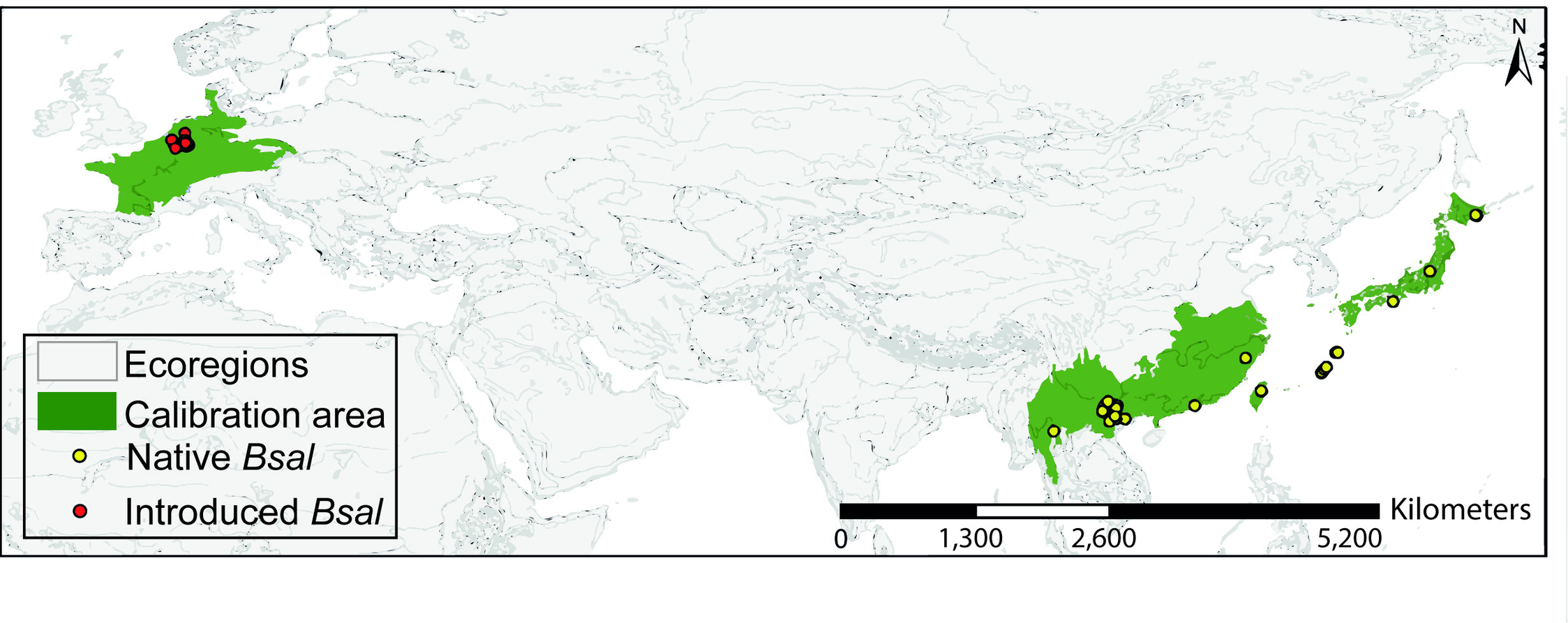

Supplement: S1 Fig — (TIF) [file pone.0211960.s001.tif]

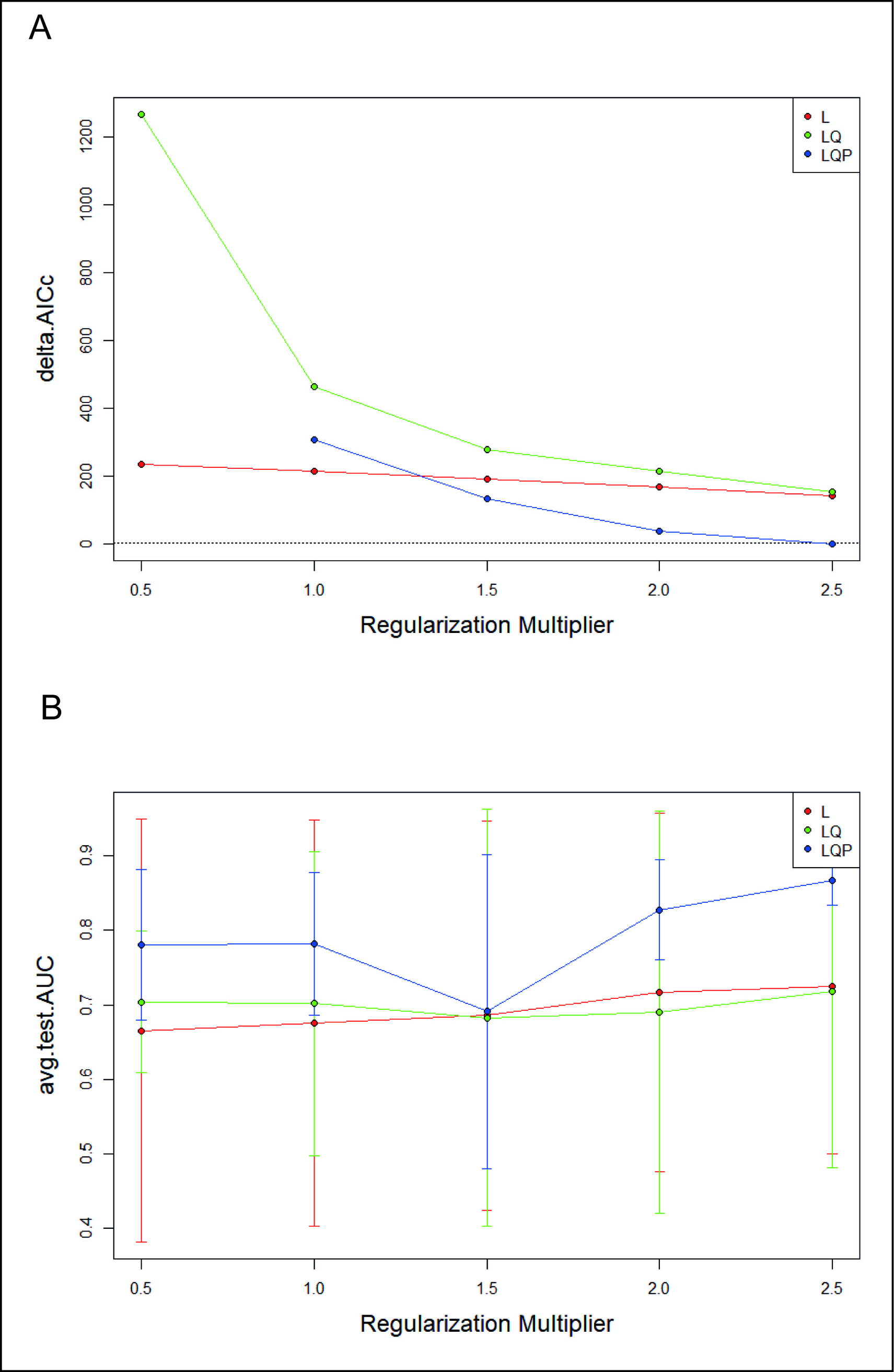

Supplement: S2 Fig — (TIF) [file pone.0211960.s002.tif]

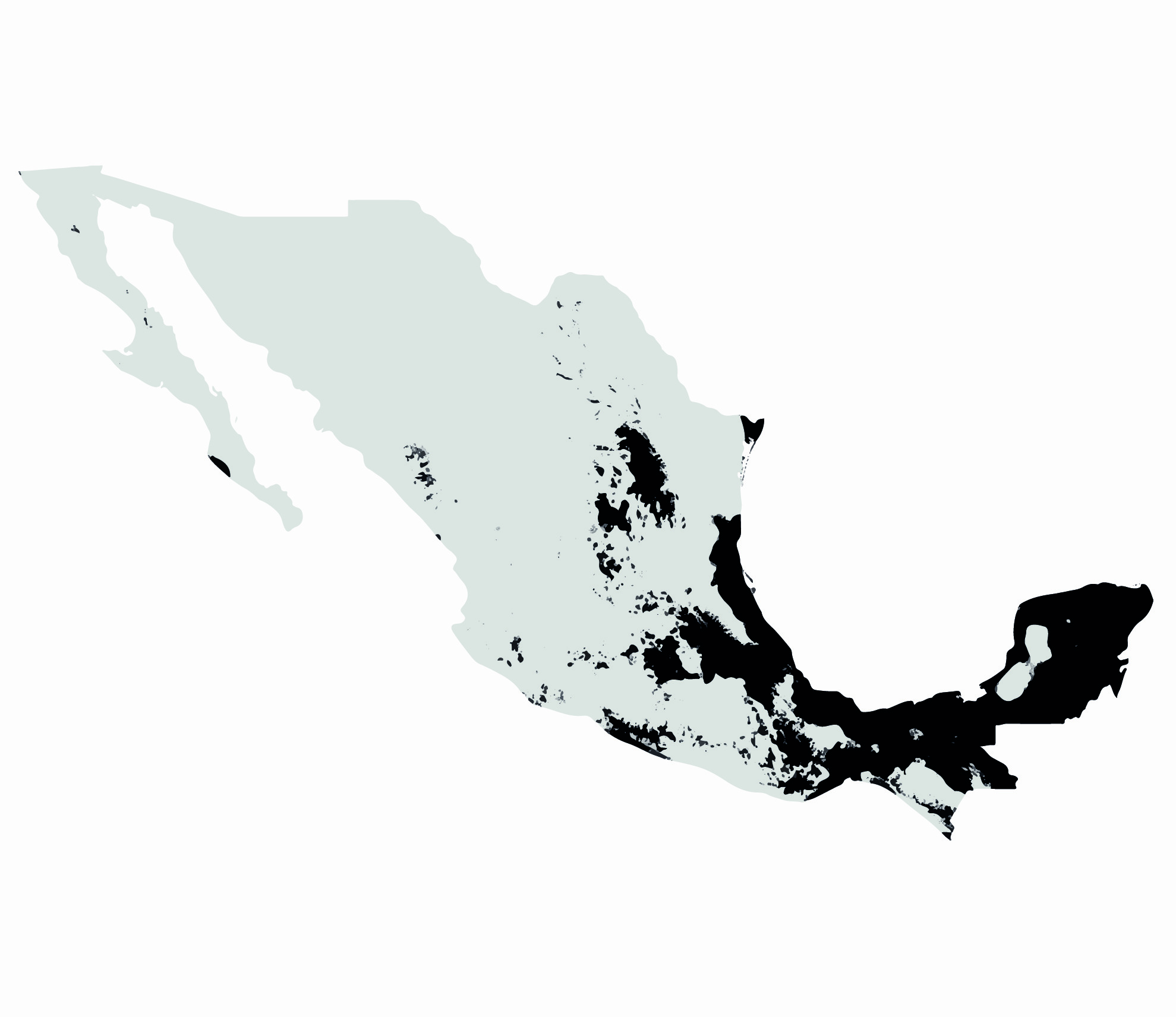

Supplement: S3 Fig — Areas of potential presence are in black, and areas of potential absence are in gray. (TIF) [file pone.0211960.s003.tif]

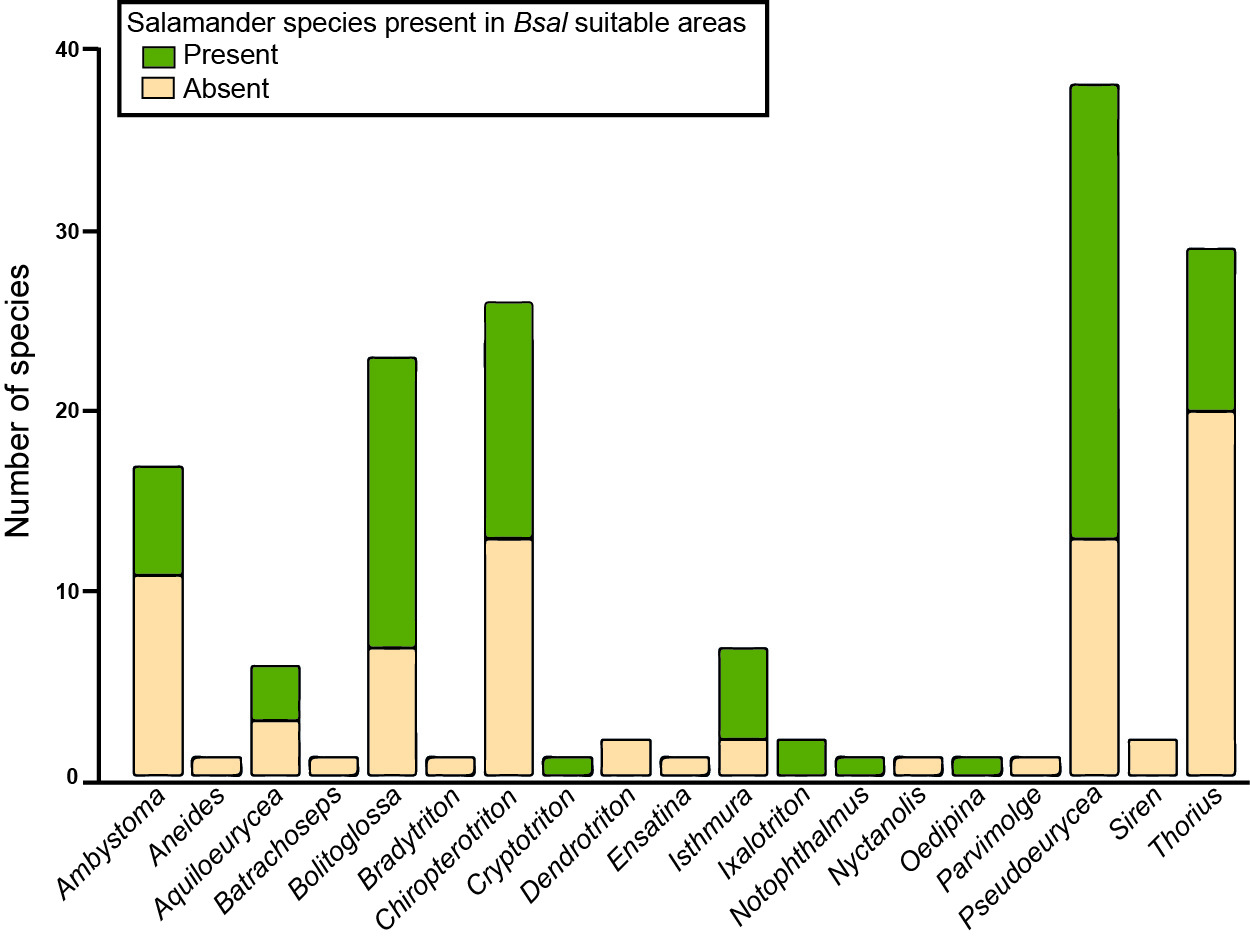

Supplement: S4 Fig — (TIF) [file pone.0211960.s004.tif]
