# Supplementary material for: Potential risk of Batrachochytrium salamandrivorans in Mexico
Source: PLoS One. 2019 Feb 12;14(2):e0211960. doi: 10.1371/journal.pone.0211960 (PMC6372179; doi:10.1371/journal.pone.0211960)
Supplement: S1 Table — (DOCX) [file pone.0211960.s005.docx]

| **Name** | **Latitude** | **Longitude** |
| --- | --- | --- |
| *Batrachochytrium salamandrivorans* (Native) | 19.87 | 99.05 |
| *Batrachochytrium salamandrivorans* (Native) | 22.62 | 105.87 |
| *Batrachochytrium salamandrivorans* (Native) | 28.27 | 129.3 |
| *Batrachochytrium salamandrivorans* (Native) | 28.34 | 129.5 |
| *Batrachochytrium salamandrivorans* (Native) | 26.14 | 127.77 |
| *Batrachochytrium salamandrivorans* (Native) | 26.18 | 127.8 |
| *Batrachochytrium salamandrivorans* (Native) | 26.54 | 128.03 |
| *Batrachochytrium salamandrivorans* (Native) | 26.75 | 128.24 |
| *Batrachochytrium salamandrivorans* (Native) | 33.79 | 135.43 |
| *Batrachochytrium salamandrivorans* (Native) | 37.02 | 139.39 |
| *Batrachochytrium salamandrivorans* (Native) | 43.02 | 144.41 |
| *Batrachochytrium salamandrivorans* (Native) | 43.08 | 144.28 |
| *Batrachochytrium salamandrivorans* (Native) | 22.54 | 104.42 |
| *Batrachochytrium salamandrivorans* (Native) | 22.29 | 105.81 |
| *Batrachochytrium salamandrivorans* (Native) | 21.18 | 106.73 |
| *Batrachochytrium salamandrivorans* (Native) | 21.18 | 106.66 |
| *Batrachochytrium salamandrivorans* (Native) | 21.20 | 105.72 |
| *Batrachochytrium salamandrivorans* (Native) | 22.37 | 105.73 |
| *Batrachochytrium salamandrivorans* (Native) | 23.03 | 104.87 |
| *Batrachochytrium salamandrivorans* (Native) | 23.04 | 104.85 |
| *Batrachochytrium salamandrivorans* (Native) | 21.96 | 104.35 |
| *Batrachochytrium salamandrivorans* (Native) | 21.96 | 104.35 |
| *Batrachochytrium salamandrivorans* (Native) | 22.00 | 104.26 |
| *Batrachochytrium salamandrivorans* (Native) | 20.94 | 105.04 |
| *Batrachochytrium salamandrivorans* (Native) | 20.94 | 105.04 |
| *Batrachochytrium salamandrivorans* (Native) | 20.92 | 105.05 |
| *Batrachochytrium salamandrivorans* (Native) | 21.46 | 105.65 |
| *Batrachochytrium salamandrivorans* (Native) | 24.15 | 121.28 |
| *Batrachochytrium salamandrivorans* (Native) | 24.16 | 121.29 |
| *Batrachochytrium salamandrivorans* (Native) | 24.19 | 121.31 |
| *Batrachochytrium salamandrivorans* (Native) | 27.72 | 119.61 |
| *Batrachochytrium salamandrivorans* (Native) | 22.6 | 114.2 |
| *Batrachochytrium salamandrivorans* (Introduced) | 50.91 | 5.74 |
| *Batrachochytrium salamandrivorans* (Introduced) | 50.63 | 6.09 |
| *Batrachochytrium salamandrivorans* (Introduced) | 50.45 | 6.1 |
| *Batrachochytrium salamandrivorans* (Introduced) | 50.58 | 5.59 |
| *Batrachochytrium salamandrivorans* (Introduced) | 50.85 | 5.97 |
| *Batrachochytrium salamandrivorans* (Introduced) | 51.82 | 5.92 |
| *Batrachochytrium salamandrivorans* (Introduced) | 50.88 | 5.75 |
| *Batrachochytrium salamandrivorans* (Introduced) | 51.09 | 4.51 |
| *Batrachochytrium salamandrivorans* (Introduced) | 50.57 | 6.28 |
| *Batrachochytrium salamandrivorans* (Introduced) | 50.7 | 6.27 |
| *Batrachochytrium salamandrivorans* (Introduced) | 50.7 | 6.27 |
| *Batrachochytrium salamandrivorans* (Introduced) | 50.9 | 6.08 |
| *Batrachochytrium salamandrivorans* (Introduced) | 51.07 | 5.92 |
| *Batrachochytrium salamandrivorans* (Introduced) | 50.22 | 4.89 |
| *Batrachochytrium salamandrivorans* (Introduced) | 50.77 | 5.95 |
