# Supplementary material for: Potential risk of Batrachochytrium salamandrivorans in Mexico
Source: PLoS One. 2019 Feb 12;14(2):e0211960. doi: 10.1371/journal.pone.0211960 (PMC6372179; doi:10.1371/journal.pone.0211960)
Supplement: S2 Table — (DOCX) [file pone.0211960.s006.docx]

| Variable | Percent contribution | Permutation importance |
| --- | --- | --- |
| Bio7 | 43.4 | 10.9 |
| Bio2 | 17 | 12.7 |
| Bio15 | 11.3 | 28.2 |
| Bio18 | 9.3 | 5.3 |
| Bio19 | 6.4 | 0.4 |
| Bio5 | 5.3 | 11.8 |
| Bio3 | 2 | 1.7 |
| Bio14 | 1.7 | 10.2 |
| Bio9 | 1.7 | 8.1 |
| Bio8 | 0.8 | 3.5 |
| Bio12 | 0.5 | 1.8 |
| Bio6 | 0.3 | 5.3 |
| Bio13 | 0.1 | 0 |
| Bio4 | 0.1 | 0.1 |
| Bio17 | 0 | 0 |
| Bio16 | 0 | 0 |
| Bio11 | 0 | 0 |
| Bio10 | 0 | 0 |
| Bio1 | 0 | 0 |
