# Supplementary material for: Potential risk of Batrachochytrium salamandrivorans in Mexico
Source: PLoS One. 2019 Feb 12;14(2):e0211960. doi: 10.1371/journal.pone.0211960 (PMC6372179; doi:10.1371/journal.pone.0211960)
Supplement: S3 Table — Variables selected with less than r = 0.75 are in bold. (DOCX) [file pone.0211960.s007.docx]

|  | **Bio19** | Bio1 | **Bio2** | Bio3 | Bio4 | **Bio5** | Bio6 | **Bio7** | Bio8 | Bio9 | Bio10 | Bio11 | Bio12 | Bio13 | Bio14 | **Bio15** | Bio16 | Bio17 | **Bio18** |
| --- | --- | --- | --- | --- | --- | --- | --- | --- | --- | --- | --- | --- | --- | --- | --- | --- | --- | --- | --- |
| **Bio19** | - | -0.43 | -0.51 | -0.55 | 0.46 | -0.20 | -0.40 | 0.29 | -0.47 | -0.22 | -0.20 | -0.50 | 0.17 | -0.20 | 0.93 | -0.65 | -0.20 | 0.94 | -0.21 |
| Bio1 | - | - | 0.26 | 0.35 | -0.37 | 0.86 | 0.91 | -0.23 | 0.73 | 0.78 | 0.87 | 0.94 | 0.55 | 0.71 | -0.55 | 0.77 | 0.71 | -0.49 | 0.58 |
| **Bio2** | - | - | - | 0.77 | -0.50 | 0.12 | 0.19 | -0.11 | 0.12 | 0.31 | 0.01 | 0.38 | -0.05 | 0.17 | -0.51 | 0.45 | 0.16 | -0.52 | 0.08 |
| Bio3 | - | - | - | - | -0.92 | -0.06 | 0.52 | -0.70 | 0.01 | 0.58 | -0.10 | 0.62 | -0.04 | 0.21 | -0.61 | 0.47 | 0.22 | -0.63 | 0.15 |
| Bio4 | - | - | - | - | - | 0.10 | -0.65 | 0.90 | 0.04 | -0.65 | 0.12 | -0.67 | 0.01 | -0.19 | 0.55 | -0.38 | -0.21 | 0.57 | -0.11 |
| **Bio5** | - | - | - | - | - | - | 0.62 | 0.27 | 0.74 | 0.52 | 0.98 | 0.65 | 0.54 | 0.60 | -0.28 | 0.57 | 0.59 | -0.20 | 0.44 |
| Bio6 | - | - | - | - | - | - | - | -0.59 | 0.50 | 0.88 | 0.64 | 0.97 | 0.39 | 0.56 | -0.54 | 0.62 | 0.57 | -0.50 | 0.43 |
| **Bio7** | - | - | - | - | - | - | - | - | 0.15 | -0.54 | 0.22 | -0.52 | 0.08 | -0.07 | 0.37 | -0.17 | -0.09 | 0.40 | -0.08 |
| Bio8 | - | - | - | - | - | - | - | - | - | 0.26 | 0.78 | 0.56 | 0.49 | 0.63 | -0.45 | 0.71 | 0.62 | -0.41 | 0.62 |
| Bio9 | - | - | - | - | - | - | - | - | - | - | 0.50 | 0.87 | 0.31 | 0.43 | -0.42 | 0.47 | 0.44 | -0.38 | 0.27 |
| Bio10 | - | - | - | - | - | - | - | - | - | - | - | 0.66 | 0.59 | 0.65 | -0.29 | 0.61 | 0.64 | -0.21 | 0.54 |
| Bio11 | - | - | - | - | - | - | - | - | - | - | - | - | 0.44 | 0.63 | -0.63 | 0.75 | 0.64 | -0.59 | 0.50 |
| Bio12 | - | - | - | - | - | - | - | - | - | - | - | - | - | 0.91 | 0.09 | 0.51 | 0.91 | 0.16 | 0.82 |
| Bio13 | - | - | - | - | - | - | - | - | - | - | - | - | - | - | -0.28 | 0.79 | 0.99 | -0.22 | 0.90 |
| Bio14 | - | - | - | - | - | - | - | - | - | - | - | - | - | - | - | -0.73 | -0.29 | 0.99 | -0.25 |
| **Bio15** | - | - | - | - | - | - | - | - | - | - | - | - | - | - | - | - | 0.79 | -0.69 | 0.74 |
| Bio16 | - | - | - | - | - | - | - | - | - | - | - | - | - | - | - | - | - | -0.22 | 0.90 |
| Bio17 | - | - | - | - | - | - | - | - | - | - | - | - | - | - | - | - | - | - | -0.20 |
| **Bio18** | - | - | - | - | - | - | - | - | - | - | - | - | - | - | - | - | - | - | - |
