# Supplementary material for: Potential risk of Batrachochytrium salamandrivorans in Mexico
Source: PLoS One. 2019 Feb 12;14(2):e0211960. doi: 10.1371/journal.pone.0211960 (PMC6372179; doi:10.1371/journal.pone.0211960)
Supplement: S4 Table — (DOCX) [file pone.0211960.s008.docx]

| Features | RM | AUCTRAIN | AUCTEST | MTPTEST | ORMTP | OR10 | AICc | ΔAICc | w.AIC | Parameters |
| --- | --- | --- | --- | --- | --- | --- | --- | --- | --- | --- |
| L | 0.5 | 0.8669 | 0.66552 | 0 | 0.09091 | 0.03306 | 1732.08 | 234.671 | 1.10E-51 | 6 |
| LQ | 0.5 | 0.8187 | 0.70386 | 0 | 0 | 0 | 2762.17 | 1264.77 | 2.29E-275 | 11 |
| LQP | 0.5 | 0.8913 | 0.78032 | 0 | 0.06818 | 0.00758 | NA | NA | NA | 18 |
| L | 1 | 0.8738 | 0.67542 | 0 | 0.09091 | 0.03306 | 1709.95 | 212.542 | 7.03E-47 | 6 |
| LQ | 1 | 0.8694 | 0.70176 | 0 | 0.04546 | 0.00826 | 1959.27 | 461.862 | 5.10E-101 | 11 |
| LQP | 1 | 0.9235 | 0.78188 | 0 | 0.02273 | 0.00207 | 1804.53 | 307.128 | 2.03E-67 | 14 |
| L | 1.5 | 0.8792 | 0.68596 | 0 | 0.09091 | 0.03306 | 1687.48 | 190.079 | 5.31E-42 | 6 |
| LQ | 1.5 | 0.9091 | 0.68238 | 0 | 0.09091 | 0.03306 | 1773.48 | 276.073 | 1.13E-60 | 10 |
| LQP | 1.5 | 0.936 | 0.69092 | 0.02273 | 0.09091 | 0.03306 | 1628.8 | 131.392 | 2.94E-29 | 9 |
| L | 2 | 0.8833 | 0.71664 | 0.04546 | 0.15909 | 0.03512 | 1663.62 | 166.218 | 8.06E-37 | 6 |
| LQ | 2 | 0.9145 | 0.69016 | 0 | 0.09091 | 0.03306 | 1710.06 | 212.657 | 6.64E-47 | 8 |
| LQP | 2 | 0.9401 | 0.82745 | 0.04546 | 0.18182 | 0.04408 | 1532.88 | 35.4786 | 1.98E-08 | 8 |
| L | 2.5 | 0.8867 | 0.72533 | 0.04546 | 0.15909 | 0.03512 | 1638.35 | 140.943 | 2.48E-31 | 6 |
| LQ | 2.5 | 0.8923 | 0.71777 | 0.04546 | 0.18182 | 0.04408 | 1649.27 | 151.866 | 1.05E-33 | 6 |
| LQP | 2.5 | 0.9397 | 0.86693 | 0.04546 | 0.15909 | 0.03512 | 1497.4 | 0 | 0.99999998 | 5 |
