# Supplementary material for: Potential risk of Batrachochytrium salamandrivorans in Mexico
Source: PLoS One. 2019 Feb 12;14(2):e0211960. doi: 10.1371/journal.pone.0211960 (PMC6372179; doi:10.1371/journal.pone.0211960)
Supplement: S5 Table — (DOCX) [file pone.0211960.s009.docx]

| Variable | Percent Contribution | Permutation Importance |
| --- | --- | --- |
| Bio7 | 42.8497 | 45.6188 |
| Bio5 | 26.2472 | 20.2716 |
| Bio15 | 22.2275 | 12.9968 |
| Bio2 | 5.7848 | 17.4223 |
| Bio18 | 1.9229 | 0 |
| Bio19 | 0.968 | 3.6905 |
