# Supplementary material for: Potential risk of Batrachochytrium salamandrivorans in Mexico
Source: PLoS One. 2019 Feb 12;14(2):e0211960. doi: 10.1371/journal.pone.0211960 (PMC6372179; doi:10.1371/journal.pone.0211960)
Supplement: S6 Table — Hotspots = pixels with Bsal suitability values greater than 0.5 and more than five salamander species. IUCN status: critically endangered (CR), endangered (E), vulnerable (V), near threatened (NT), least concern (LC), data deficient (DD), and not evaluated (NE). (DOCX) [file pone.0211960.s010.docx]

| \| **Species** \| **Genus** \| **Range km2** \| **Range categories** \| **IUCN status** \| **Endemic to Mexico** \| **Presence in Hotspot** \| **Area** \| **Hotspot** \| ***Bsal* suitability range** \| **Distribution in *Bsal* suitable areas** \| \| --- \| --- \| --- \| --- \| --- \| --- \| --- \| --- \| --- \| --- \| --- \| \| *Ambystoma altamirani* \| *Ambystoma* \| 1613 \| 1000-5000 \| E \| Yes \| Yes \| TVB \| 3 \| 0-0.56 \| Yes \| \| *Ambystoma amblycephalum* \| *Ambystoma* \| 20 \| <100 \| CR \| Yes \| No \| - \| - \| 0.01-0.12 \| No \| \| *Ambystoma andersoni* \| *Ambystoma* \| 21 \| <100 \| CR \| Yes \| No \| - \| - \| 0-0.02 \| No \| \| *Ambystoma bombypellum* \| *Ambystoma* \| 96 \| <100 \| CR \| Yes \| No \| - \| - \| 0.04-0.19 \| No \| \| *Ambystoma dumerilii* \| *Ambystoma* \| 6 \| <100 \| CR \| Yes \| No \| - \| - \| 0 \| No \| \| *Ambystoma flavipiperatum* \| *Ambystoma* \| 257 \| 100-500 \| E \| Yes \| No \| - \| - \| 0-0.04 \| No \| \| *Ambystoma granulosum* \| *Ambystoma* \| 8937 \| >5000 \| CR \| Yes \| Yes \| TVB \| 1, 2 \| 0-0.7 \| Yes \| \| *Ambystoma leorae* \| *Ambystoma* \| 39 \| <100 \| CR \| Yes \| Yes \| TVB \| 6 \| 0.16-0.5 \| Yes \| \| *Ambystoma lermaense* \| *Ambystoma* \| 90 \| <100 \| E \| Yes \| No \| - \| - \| 0-0.18 \| No \| \| *Ambystoma mavortium* \| *Ambystoma* \| 210648 \| >5000 \| LC \| No \| No \| - \| - \| 0-0.15 \| No \| \| *Ambystoma mexicanum* \| *Ambystoma* \| 114 \| 100-500 \| CR \| Yes \| No \| - \| - \| 0-0.38 \| No \| \| *Ambystoma ordinarium* \| *Ambystoma* \| 4928 \| 1000-5000 \| E \| Yes \| No \| - \| - \| 0-0.53 \| Yes \| \| *Ambystoma rivulare* \| *Ambystoma* \| 16955 \| >5000 \| DD \| Yes \| Yes \| TVB \| 1-3 \| 0-0.89 \| Yes \| \| *Ambystoma rosaceum* \| *Ambystoma* \| 204227 \| >5000 \| LC \| Yes \| No \|  \|  \| 0-0.13 \| No \| \| *Ambystoma silvense* \| *Ambystoma* \| 1276 \| 1000-5000 \| DD \| Yes \| No \|  \|  \| 0-0.04 \| No \| \| *Ambystoma taylori* \| *Ambystoma* \| 17 \| <100 \| CR \| Yes \| No \|  \|  \| 0.19-0.4 \| No \| \| *Ambystoma velasci* \| *Ambystoma* \| 823141 \| >5000 \| LC \| Yes \| Yes \| TVB \| 1, 2, 4-6 \| 0-0.95 \| Yes \| \| *Aneides lugubris* \| *Aneides* \| 12968 \| >5000 \| LC \| No \| No \|  \|  \| 0-0.06 \| No \| \| *Aquiloerycea cephalica* \| *Aquiloeurycea* \| 18726 \| >5000 \| NT \| Yes \| Yes \| TVB \| 3, 4-8 \| 0-0.95 \| Yes \| \| *Aquiloeurycea cafetalera* \| *Aquiloeurycea* \| 632 \| 500-1000 \| NE \| Yes \| Yes \| TVB \| 8 \| 0.07-0.53 \| Yes \| \| *Aquiloeurycea galeanae* \| *Aquiloeurycea* \| 1090 \| 1000-5000 \| V \| Yes \| No \|  \|  \| 0.02-0.7 \| Yes \| \| *Aquiloeurycea praecellens* \| *Aquiloeurycea* \| 25 \| <100 \| CR \| Yes \| No \|  \|  \| 0.02-0.08 \| No \| \| *Aquiloeurycea quetzalanensis* \| *Aquiloeurycea* \| 60 \| <100 \| CR \| Yes \| No \|  \|  \| 0.01-0.24 \| No \| \| *Aquiloeurycea scandens* \| *Aquiloeurycea* \| 1247 \| 1000-5000 \| LC \| Yes \| No \|  \|  \| 0-0.08 \| No \| \| *Batrachoseps major* \| *Batrachoseps* \| 17085 \| >5000 \| LC \| N0 \| No \|  \|  \| 0-0.07 \| No \| \| *Bolitoglossa alberchi* \| *Bolitoglossa* \| 6100 \| >5000 \| LC \| Yes \| Yes \| Los Tuxtlas \| 9, 10 \| 0.02-0.76 \| Yes \| \| *Bolitoglossa chinanteca* \| *Bolitoglossa* \| 281 \| 100-500 \| NE \| Yes \| No \|  \|  \| 0-0.65 \| Yes \| \| *Bolitoglossa engelhardti* \| *Bolitoglossa* \| 135 \| 100-500 \| E \| No \| Yes \| SMS Chiapas \| 13 \| 0.02-0.73 \| Yes \| \| *Bolitoglossa flavimembris* \| *Bolitoglossa* \| 492 \| 100-500 \| E \| No \| Yes \| SMS Chiapas \| 13 \| 0-0.73 \| Yes \| \| *Bolitoglossa flaviventris* \| *Bolitoglossa* \| 696 \| 500-1000 \| E \| No \| No \|  \|  \| 0-0.15 \| No \| \| *Bolitoglossa franklini* \| *Bolitoglossa* \| 2391 \| 1000-5000 \| E \| No \| Yes \| SMS Chiapas \| 13 \| 0-0.73 \| Yes \| \| *Bolitoglossa hartwegi* \| *Bolitoglossa* \| 1565 \| 1000-5000 \| NT \| No \| No \|  \|  \| 0.02-0.74 \| Yes \| \| *Bolitoglossa hermosa* \| *Bolitoglossa* \| 376 \| 100-500 \| NT \| Yes \| No \|  \|  \| 0.02-0.76 \| Yes \| \| *Bolitoglossa lincolni* \| *Bolitoglossa* \| 1124 \| 1000-5000 \| NT \| No \| No \|  \|  \| 0.05-0.74 \| Yes \| \| *Bolitoglossa macrinii* \| *Bolitoglossa* \| 4701 \| 1000-5000 \| NT \| Yes \| No \|  \|  \| 0-0.79 \| Yes \| \| *Bolitoglossa mexicana* \| *Bolitoglossa* \| 34021 \| >5000 \| LC \| No \| No \|  \|  \| 0-0.78 \| Yes \| \| *Bolitoglossa mulleri* \| *Bolitoglossa* \| 75 \| <100 \| V \| No \| No \|  \|  \| 0.02-0.04 \| No \| \| *Bolitoglossa oaxacensis* \| *Bolitoglossa* \| 1776 \| 1000-5000 \| DD \| Yes \| No \|  \|  \| 0-0.31 \| No \| \| *Bolitoglossa occidentalis* \| *Bolitoglossa* \| 7756 \| >5000 \| LC \| No \| Yes \| SMS Chiapas \| 13 \| 0-0.63 \| No \| \| *Bolitoglossa odonnelli* \| *Bolitoglossa* \| 2 \| <100 \| NT \| No \| No \|  \|  \| 0.15-0.22 \| No \| \| *Bolitoglossa platydactyla* \| *Bolitoglossa* \| 63335 \| >5000 \| NT \| Yes \| Yes \| Los Tuxtlas \| 9, 10 \| 0-0.88 \| Yes \| \| *Bolitoglossa riletti* \| *Bolitoglossa* \| 331 \| 100-500 \| E \| Yes \| No \|  \|  \| 0 \| No \| \| *Bolitoglossa rostrata* \| *Bolitoglossa* \| 70 \| <100 \| V \| No \| Yes \| SMS Chiapas \| 13 \| 0.03-0.73 \| No \| \| *Bolitoglossa rufescens* \| *Bolitoglossa* \| 47833 \| >5000 \| LC \| No \| Yes \| Los Tuxtlas \| 9, 10 \| 0-0.78 \| Yes \| \| *Bolitoglossa stuarti* \| *Bolitoglossa* \| 2442 \| 1000-5000 \| DD \| No \| No \|  \|  \| 0-0.55 \| Yes \| \| *Bolitoglossa veracrucis* \| *Bolitoglossa* \| 4821 \| 1000-5000 \| E \| Yes \| No \|  \|  \| 0.02-0.66 \| Yes \| \| *Bolitoglossa yucatana* \| *Bolitoglossa* \| 52512 \| >5000 \| LC \| No \| No \|  \|  \| 0.02-0.95 \| Yes \| \| *Bolitoglossa zapoteca* \| *Bolitoglossa* \| 83 \| <100 \| CR \| Yes \| No \|  \|  \| 0.02-0.72 \| Yes \| \| *Bradytriton silus* \| *Bradytriton* \| 3 \| <100 \| CR \| No \| No \|  \|  \| 0.23-0.33 \| No \| \| *Chiropterotriton arboreus* \| *Chiropterotriton* \| 15 \| <100 \| CR \| Yes \| No \|  \|  \| 0.07-0.12 \| No \| \| *Chiropterotriton chico* \| *Chiropterotriton* \| 3 \| <100 \| NE \| Yes \| Yes \| TVB \| 4 \| 0.56-0.6 \| Yes \| \| *Chiropterotriton aureus* \| *Chiropterotriton* \| 3 \| <100 \| NE \| Yes \| No \|  \|  \| 0.44-0.53 \| Yes \| \| *Chiropterotriton chiropterus* \| *Chiropterotriton* \| 100 \| 100-500 \| CR \| Yes \| No \|  \|  \| 0.14-0.32 \| No \| \| *Chiropterotriton chondrostega* \| *Chiropterotriton* \| 778 \| 500-1000 \| E \| Yes \| No \|  \|  \| 0-0.56 \| Yes \| \| *Chiropterotriton cieloensis* \| *Chiropterotriton* \| 3 \| <100 \| NE \| Yes \| No \|  \|  \| 0.01-0.01 \| No \| \| *Chiropterotriton cracens* \| *Chiropterotriton* \| 49 \| <100 \| E \| Yes \| No \|  \|  \| 0-0.03 \| No \| \| *Chiropterotriton dimidiatus* \| *Chiropterotriton* \| 255 \| 100-500 \| E \| Yes \| Yes \| TVB \| 4 \| 0.09-0.65 \| Yes \| \| *Chiropterotriton infernalis* \| *Chiropterotriton* \| 15 \| <100 \| NE \| Yes \| No \|  \|  \| 0-0.01 \| No \| \| *Chiropterotriton lavae* \| *Chiropterotriton* \| 81 \| <100 \| CR \| Yes \| Yes \| TVB \| 8 \| 0.3-0.62 \| Yes \| \| *Chiropterotriton magnipes* \| *Chiropterotriton* \| 403 \| 100-500 \| CR \| Yes \| No \|  \|  \| 0-0.15 \| No \| \| *Chiropterotriton miquihuanus* \| *Chiropterotriton* \| 94 \| <100 \| NE \| Yes \| No \|  \|  \| 0.11-0.72 \| Yes \| \| *Chiropterotriton mosaueri* \| *Chiropterotriton* \| 46 \| <100 \| CR \| Yes \| No \|  \|  \| 0.03-0.1 \| No \| \| *Chiropterotriton multidentatus* \| *Chiropterotriton* \| 4558 \| 1000-5000 \| E \| Yes \| No \|  \|  \| 0-0.59 \| Yes \| \| *Chiropterotriton nubilus* \| *Chiropterotriton* \| 21 \| <100 \| NE \| Yes \| No \|  \|  \| 0.14-0.68 \| Yes \| \| *Chiropterotriton orculus* \| *Chiropterotriton* \| 10051 \| >5000 \| V \| Yes \| Yes \| TVB \| 1, 3, 5, 6 \| 0-0.95 \| Yes \| \| *Chiropterotriton priscus* \| *Chiropterotriton* \| 3166 \| 1000-5000 \| NT \| Yes \| No \|  \|  \| 0-0.89 \| Yes \| \| *Chiropterotriton sp C* \| *Chiropterotriton* \| 3 \| <100 \| NE \| Yes \| No \|  \|  \| 0.12-0.14 \| No \| \| *Chiropterotriton sp E* \| *Chiropterotriton* \| 3 \| <100 \| NE \| Yes \| Yes \| TVB \| 8 \| 0.5-0.54 \| Yes \| \| *Chiropterotriton sp F* \| *Chiropterotriton* \| 3 \| <100 \| NE \| Yes \| No \|  \|  \| 0 \| No \| \| *Chiropterotriton sp G* \| *Chiropterotriton* \| 3 \| <100 \| NE \| Yes \| No \|  \|  \| 0.2-0.27 \| No \| \| *Chiropterotriton sp H* \| *Chiropterotriton* \| 3 \| <100 \| NE \| Yes \| No \|  \|  \| 0.42-0.51 \| Yes \| \| *Chiropterotriton sp I* \| *Chiropterotriton* \| 3 \| <100 \| NE \| Yes \| No \|  \|  \| 0.31-0.36 \| No \| \| *Chiropterotriton sp J* \| *Chiropterotriton* \| 3 \| <100 \| NE \| Yes \| No \|  \|  \| 0.12-0.15 \| No \| \| *Chiropterotriton sp K* \| *Chiropterotriton* \| 3 \| <100 \| NE \| Yes \| Yes \| Northern Oaxaca \| 12 \| 0.41-0.6 \| Yes \| \| *Chiropterotriton terrestris* \| *Chiropterotriton* \| 35 \| <100 \| CR \| Yes \| No \|  \|  \| 0.06-0.14 \| No \| \| *Cryptotriton alvarezdeltoroi* \| *Cryptotriton* \| 50 \| <100 \| E \| Yes \| No \|  \|  \| 0.03-0.6 \| Yes \| \| *Dendrotriton megarhinus* \| *Dendrotriton* \| 44 \| <100 \| V \| Yes \| No \|  \|  \| 0.02-0.37 \| No \| \| *Dendrotriton xolocalcae* \| *Dendrotriton* \| 8 \| <100 \| V \| Yes \| No \|  \|  \| 0-0.05 \| No \| \| *Ensatina eschscholtzii* \| *Ensatina* \| 7534 \| >5000 \| LC \| No \| No \|  \|  \| 0-0.17 \| No \| \| *Isthmura bellii* \| *Isthmura* \| 199503 \| >5000 \| V \| Yes \| Yes \| TVB/SMS Guerrero \| 1-3, 5, 6, 11 \| 0-0.95 \| Yes \| \| *Isthmura boneti* \| *Isthmura* \| 4478 \| 1000-5000 \| V \| Yes \| Yes \| Northern Oaxaca \| 12 \| 0-0.86 \| Yes \| \| *Isthmura corrugata* \| *Isthmura* \| 3 \| <100 \| NE \| Yes \| No \|  \|  \| 0.2-0.24 \| No \| \| *Isthmura gigantea* \| *Isthmura* \| 5169 \| >5000 \| CR \| Yes \| Yes \| TVB \| 8 \| 0-0.87 \| Yes \| \| *Isthmura maxima* \| *Isthmura* \| 295 \| 100-500 \| E \| Yes \| No \|  \|  \| 0-0.5 \| Yes \| \| *Isthmura naucampatepetl* \| *Isthmura* \| 66 \| <100 \| CR \| Yes \| No \|  \|  \| 0.22-0.55 \| Yes \| \| *Isthmura sierraoccidentalis* \| *Isthmura* \| 5010 \| >5000 \| NE \| Yes \| No \|  \|  \| 0 \| No \| \| *Ixalotriton niger* \| *Ixalotriton* \| 7 \| <100 \| E \| Yes \| No \|  \|  \| 0.05-0.06 \| Yes \| \| *Ixalotriton parvus* \| *Ixalotriton* \| 14 \| <100 \| CR \| Yes \| No \|  \|  \| 0.28-0.6 \| Yes \| \| *Notophthalmus meridionalis* \| *Notophthalmus* \| 76147 \| >5000 \| E \| No \| No \|  \|  \| 0-0.56 \| Yes \| \| *Nyctanolis pernix* \| *Nyctanolis* \| 48 \| <100 \| E \| No \| No \|  \|  \| 0.02-0.1 \| No \| \| *Oedipina elongata* \| *Oedipina* \| 2512 \| 1000-5000 \| LC \| No \| No \|  \|  \| 0.02-0.71 \| Yes \| \| *Parvimolge townsendi* \| *Parvimolge* \| 770 \| 500-1000 \| CR \| Yes \| No \|  \|  \| 0.06-0.38 \| No \| \| *Pseudoeurycea ahuitzotl* \| *Pseudoeurycea* \| 53 \| <100 \| CR \| Yes \| Yes \| SMS Guerrero \| 11 \| 0.05-0.88 \| Yes \| \| *Pseudoeurycea altamontana* \| *Pseudoeurycea* \| 701 \| 500-1000 \| E \| Yes \| No \|  \|  \| 0.01-0.38 \| No \| \| *Pseudoeurycea amuzga* \| *Pseudoeurycea* \| 31 \| <100 \| DD \| Yes \| No \|  \|  \| 0 \| No \| \| *Pseudoeurycea anitae* \| *Pseudoeurycea* \| 27 \| <100 \| CR \| Yes \| No \|  \|  \| 0.02-0.16 \| No \| \| *Pseudoeurycea aquatica* \| *Pseudoeurycea* \| 10 \| <100 \| CR \| Yes \| No \|  \|  \| 0.3-0.66 \| Yes \| \| *Pseudoeurycea aurantia* \| *Pseudoeurycea* \| 37 \| <100 \| CR \| Yes \| No \|  \|  \| 0.09-0.24 \| No \| \| *Pseudoeurycea brunnata* \| *Pseudoeurycea* \| 15 \| <100 \| CR \| No \| Yes \| SMS Chiapas \| 13 \| 0.19-0.73 \| Yes \| \| *Pseudoeurycea cochranae* \| *Pseudoeurycea* \| 1803 \| 1000-5000 \| V \| Yes \| No \|  \|  \| 0-0.85 \| Yes \| \| *Pseudoeurycea conanti* \| *Pseudoeurycea* \| 51 \| <100 \| E \| Yes \| No \|  \|  \| 0-0.6 \| Yes \| \| *Pseudoeurycea firscheini* \| *Pseudoeurycea* \| 169 \| 100-500 \| E \| Yes \| No \|  \|  \| 0.1-0.3 \| No \| \| *Pseudoeurycea gadovii* \| *Pseudoeurycea* \| 2072 \| 1000-5000 \| V \| Yes \| Yes \| TVB \| 7 \| 0.04-0.95 \| Yes \| \| *Pseudoeurycea goebeli* \| *Pseudoeurycea* \| 342 \| 100-500 \| CR \| No \| Yes \| SMS Chiapas \| 13 \| 0.05-0.73 \| Yes \| \| *Pseudoeurycea juarezi* \| *Pseudoeurycea* \| 150 \| 100-500 \| CR \| Yes \| No \|  \|  \| 0-0.85 \| Yes \| \| *Pseudoeurycea kuautli* \| *Pseudoeurycea* \| 12 \| <100 \| CR \| Yes \| No \|  \|  \| 0.06-0.27 \| No \| \| *Pseudoeurycea leprosa* \| *Pseudoeurycea* \| 13509 \| >5000 \| LC \| Yes \| Yes \| TVB \| 1-4, 5-8 \| 0-0.95 \| Yes \| \| *Pseudoeurycea lineola* \| *Pseudoeurycea* \| 564 \| 500-1000 \| E \| Yes \| No \|  \|  \| 0.06-0.36 \| No \| \| *Pseudoeurycea longicauda* \| *Pseudoeurycea* \| 323 \| 100-500 \| E \| Yes \| No \|  \|  \| 0.02-0.46 \| No \| \| *Pseudoeurycea lynchi* \| *Pseudoeurycea* \| 306 \| 100-500 \| E \| Yes \| Yes \| TVB \| 7, 8 \| 0.01-0.86 \| Yes \| \| *Pseudoeurycea melanomolga* \| *Pseudoeurycea* \| 388 \| 100-500 \| E \| Yes \| Yes \| TVB \| 7 \| 0.09-0.8 \| Yes \| \| *Pseudoeurycea mixcoatl* \| *Pseudoeurycea* \| 200 \| 100-500 \| DD \| Yes \| No \|  \|  \| 0-0.6 \| Yes \| \| *Pseudoeurycea mixteca* \| *Pseudoeurycea* \| 106 \| 100-500 \| V \| Yes \| No \|  \|  \| 0-0.07 \| No \| \| *Pseudoeurycea mystax* \| *Pseudoeurycea* \| 14 \| <100 \| CR \| Yes \| No \|  \|  \| 0.23-0.76 \| Yes \| \| *Pseudoeurycea nigromaculata* \| *Pseudoeurycea* \| 135 \| 100-500 \| E \| Yes \| No \|  \|  \| 0.04-0.45 \| No \| \| *Pseudoeurycea obesa* \| *Pseudoeurycea* \| 10 \| <100 \| CR \| Yes \| No \|  \|  \| 0.05-0.09 \| No \| \| *Pseudoeurycea orchileucos* \| *Pseudoeurycea* \| 102 \| 100-500 \| CR \| Yes \| No \|  \|  \| 0-0.08 \| Yes \| \| *Pseudoeurycea orchimelas* \| *Pseudoeurycea* \| 1394 \| 1000-5000 \| E \| Yes \| Yes \| Los Tuxtlas \| 9, 10 \| 0.06-0.76 \| Yes \| \| *Pseudoeurycea papenfussi* \| *Pseudoeurycea* \| 583 \| 500-1000 \| E \| Yes \| No \|  \|  \| 0.04-0.52 \| Yes \| \| *Pseudoeurycea rex* \| *Pseudoeurycea* \| 121 \| 100-500 \| CR \| No \| Yes \| SMS Chiapas \| 13 \| 0.09-0.73 \| Yes \| \| *Pseudoeurycea robertsi* \| *Pseudoeurycea* \| 17 \| <100 \| CR \| Yes \| Yes \| TVB \| 1 \| 0.44-0.81 \| Yes \| \| *Pseudoeurycea ruficauda* \| *Pseudoeurycea* \| 11 \| <100 \| E \| Yes \| No \|  \|  \| 0.03-0.09 \| No \| \| *Pseudoeurycea saltator* \| *Pseudoeurycea* \| 7 \| <100 \| CR \| Yes \| No \|  \|  \| 0.13-0.17 \| No \| \| *Pseudoeurycea smithi* \| *Pseudoeurycea* \| 265 \| 100-500 \| CR \| Yes \| Yes \| Northern Oaxaca \| 12 \| 0.04-0.64 \| Yes \| \| *Pseudoeurycea tenchalli* \| *Pseudoeurycea* \| 99 \| <100 \| E \| Yes \| Yes \| SMS Guerrero \| 11 \| 0.03-0.85 \| Yes \| \| *Pseudoeurycea teotepec* \| *Pseudoeurycea* \| 107 \| 100-500 \| E \| Yes \| Yes \| SMS Guerrero \| 11 \| 0.03-0.88 \| Yes \| \| *Pseudoeurycea tlahcuiloh* \| *Pseudoeurycea* \| 50 \| <100 \| CR \| Yes \| Yes \| SMS Guerrero \| 11 \| 0.05-0.83 \| Yes \| \| *Pseudoeurycea tlilicxitl* \| *Pseudoeurycea* \| 1773 \| 1000-5000 \| E \| Yes \| Yes \| TVB \| 3, 6 \| 0-0.68 \| Yes \| \| *Pseudoeurycea unguidentis* \| *Pseudoeurycea* \| 45 \| <100 \| CR \| Yes \| Yes \| Northern Oaxaca \| 12 \| 0.16-0.64 \| Yes \| \| *Pseudoeurycea werleri* \| *Pseudoeurycea* \| 218 \| 100-500 \| E \| Yes \| Yes \| Los Tuxtlas \| 9, 10 \| 0.02-0.76 \| Yes \| \| *Siren intermedia* \| *Siren* \| 15457 \| >5000 \| LC \| Yes \| No \|  \|  \| 0-0.21 \| No \| \| *Siren lacertina* \| *Siren* \| 3713 \| 1000-5000 \| LC \| No \| No \|  \|  \| 0.02-0.2 \| No \| \| *Thorius adelos* \| *Thorius* \| 67 \| <100 \| E \| Yes \| No \|  \|  \| 0.04-0.29 \| No \| \| *Thorius arboreus* \| *Thorius* \| 26 \| <100 \| E \| Yes \| No \|  \|  \| 0.01-0.29 \| No \| \| *Thorius aureus* \| *Thorius* \| 17 \| <100 \| CR \| Yes \| No \|  \|  \| 0.15-0.36 \| No \| \| *Thorius boreas* \| *Thorius* \| 154 \| 100-500 \| E \| Yes \| No \|  \|  \| 0-0.45 \| No \| \| *Thorius dubitus* \| *Thorius* \| 92 \| <100 \| E \| Yes \| No \|  \|  \| 0.1-0.3 \| No \| \| *Thorius grandis* \| *Thorius* \| 40 \| <100 \| E \| Yes \| Yes \| SMS Guerrero \| 11 \| 0.05-0.9 \| Yes \| \| *Thorius hankeni* \| *Thorius* \| 3 \| <100 \| NE \| Yes \| No \|  \|  \| 0.35-0.47 \| No \| \| *Thorius infernalis* \| *Thorius* \| 35 \| <100 \| CR \| Yes \| No \|  \|  \| 0.02-0.04 \| No \| \| *Thorius insperatus* \| *Thorius* \| 19 \| <100 \| CR \| Yes \| No \|  \|  \| 0.04-0.08 \| No \| \| *Thorius longicaudus* \| *Thorius* \| 3 \| <100 \| CR \| Yes \| No \|  \|  \| 0.02-0.04 \| No \| \| *Thorius lunaris* \| *Thorius* \| 211 \| 100-500 \| E \| Yes \| No \|  \|  \| 0.09-0.34 \| No \| \| *Thorius macdougalli* \| *Thorius* \| 555 \| 500-1000 \| V \| Yes \| No \|  \|  \| 0-0.81 \| Yes \| \| *Thorius magnipes* \| *Thorius* \| 46 \| <100 \| CR \| Yes \| No \|  \|  \| 0.1-0.17 \| No \| \| *Thorius maxillabrochus* \| *Thorius* \| 3 \| <100 \| NE \| Yes \| No \|  \|  \| 0.06-0.1 \| No \| \| *Thorius minutissimus* \| *Thorius* \| 55 \| <100 \| CR \| Yes \| No \|  \|  \| 0.02-0.7 \| No \| \| *Thorius minydemus* \| *Thorius* \| 146 \| 100-500 \| E \| Yes \| Yes \| TVB \| 8 \| 0.33-0.86 \| Yes \| \| *Thorius munificus* \| *Thorius* \| 43 \| <100 \| CR \| Yes \| Yes \| TVB \| 8 \| 0.39-0.57 \| Yes \| \| *Thorius narismagnus* \| *Thorius* \| 21 \| <100 \| CR \| Yes \| Yes \| Los Tuxtlas \| 9 \| 0.55-0.72 \| Yes \| \| *Thorius narisovalis* \| *Thorius* \| 84 \| <100 \| CR \| Yes \| Yes \| Northern Oaxaca \| 12 \| 0.05-0.79 \| Yes \| \| *Thorius omiltemi* \| *Thorius* \| 40 \| <100 \| E \| Yes \| No \|  \|  \| 0.01-0.77 \| Yes \| \| *Thorius papaloae* \| *Thorius* \| 137 \| 100-500 \| E \| Yes \| No \|  \|  \| 0.04-0.3 \| No \| \| *Thorius pennatulus* \| *Thorius* \| 811 \| 500-1000 \| CR \| Yes \| No \|  \|  \| 0.03-0.41 \| No \| \| *Thorius pinicola* \| *Thorius* \| 3 \| <100 \| CR \| Yes \| No \|  \|  \| 0.66-0.74 \| Yes \| \| *Thorius pulmonaris* \| *Thorius* \| 50 \| <100 \| E \| Yes \| Yes \| Northern Oaxaca \| 12 \| 0.1-0.6 \| Yes \| \| *Thorius schmidti* \| *Thorius* \| 217 \| 100-500 \| E \| Yes \| No \|  \|  \| 0-0.3 \| No \| \| *Thorius smithi* \| *Thorius* \| 9 \| <100 \| CR \| Yes \| No \|  \|  \| 0.04-0.08 \| No \| \| *Thorius spilogaster* \| *Thorius* \| 34 \| <100 \| CR \| Yes \| No \|  \|  \| 0.1-0.3 \| No \| \| *Thorius tlaxiacus* \| *Thorius* \| 3 \| <100 \| CR \| Yes \| No \|  \|  \| 0.04-0.09 \| No \| \| *Thorius troglodytes* \| *Thorius* \| 194 \| 100-500 \| E \| Yes \| No \|  \|  \| 0.1-0.4 \| No \| |  |  |  |  |  |  |  |  |  |  |  |  |  |  |  |  |  |  |  |
| --- | --- | --- | --- | --- | --- | --- | --- | --- | --- | --- | --- | --- | --- | --- | --- | --- | --- | --- | --- | --- | --- | --- | --- | --- | --- | --- | --- | --- | --- | --- | --- | --- | --- | --- | --- | --- | --- | --- | --- | --- | --- | --- | --- | --- | --- | --- | --- | --- | --- | --- | --- | --- | --- | --- | --- | --- | --- | --- | --- | --- | --- | --- | --- | --- | --- | --- | --- | --- | --- | --- | --- | --- | --- | --- | --- | --- | --- | --- | --- | --- | --- | --- | --- | --- | --- | --- | --- | --- | --- | --- | --- | --- | --- | --- | --- | --- | --- | --- | --- | --- | --- | --- | --- | --- | --- | --- | --- | --- | --- | --- | --- | --- | --- | --- | --- | --- | --- | --- | --- | --- | --- | --- | --- | --- | --- | --- | --- | --- | --- | --- | --- | --- | --- | --- | --- | --- | --- | --- | --- | --- | --- | --- | --- | --- | --- | --- | --- | --- | --- | --- | --- | --- | --- | --- | --- | --- | --- | --- | --- | --- | --- | --- | --- | --- | --- | --- | --- | --- | --- | --- | --- | --- | --- | --- | --- | --- | --- | --- | --- | --- | --- | --- | --- | --- | --- | --- | --- | --- | --- | --- | --- | --- | --- | --- | --- | --- | --- | --- | --- | --- | --- | --- | --- | --- | --- | --- | --- | --- | --- | --- | --- | --- | --- | --- | --- | --- | --- | --- | --- | --- | --- | --- | --- | --- | --- | --- | --- | --- | --- | --- | --- | --- | --- | --- | --- | --- | --- | --- | --- | --- | --- | --- | --- | --- | --- | --- | --- | --- | --- | --- | --- | --- | --- | --- | --- | --- | --- | --- | --- | --- | --- | --- | --- | --- | --- | --- | --- | --- | --- | --- | --- | --- | --- | --- | --- | --- | --- | --- | --- | --- | --- | --- | --- | --- | --- | --- | --- | --- | --- | --- | --- | --- | --- | --- | --- | --- | --- | --- | --- | --- | --- | --- | --- | --- | --- | --- | --- | --- | --- | --- | --- | --- | --- | --- | --- | --- | --- | --- | --- | --- | --- | --- | --- | --- | --- | --- | --- | --- | --- | --- | --- | --- | --- | --- | --- | --- | --- | --- | --- | --- | --- | --- | --- | --- | --- | --- | --- | --- | --- | --- | --- | --- | --- | --- | --- | --- | --- | --- | --- | --- | --- | --- | --- | --- | --- | --- | --- | --- | --- | --- | --- | --- | --- | --- | --- | --- | --- | --- | --- | --- | --- | --- | --- | --- | --- | --- | --- | --- | --- | --- | --- | --- | --- | --- | --- | --- | --- | --- | --- | --- | --- | --- | --- | --- | --- | --- | --- | --- | --- | --- | --- | --- | --- | --- | --- | --- | --- | --- | --- | --- | --- | --- | --- | --- | --- | --- | --- | --- | --- | --- | --- | --- | --- | --- | --- | --- | --- | --- | --- | --- | --- | --- | --- | --- | --- | --- | --- | --- | --- | --- | --- | --- | --- | --- | --- | --- | --- | --- | --- | --- | --- | --- | --- | --- | --- | --- | --- | --- | --- | --- | --- | --- | --- | --- | --- | --- | --- | --- | --- | --- | --- | --- | --- | --- | --- | --- | --- | --- | --- | --- | --- | --- | --- | --- | --- | --- | --- | --- | --- | --- | --- | --- | --- | --- | --- | --- | --- | --- | --- | --- | --- | --- | --- | --- | --- | --- | --- | --- | --- | --- | --- | --- | --- | --- | --- | --- | --- | --- | --- | --- | --- | --- | --- | --- | --- | --- | --- | --- | --- | --- | --- | --- | --- | --- | --- | --- | --- | --- | --- | --- | --- | --- | --- | --- | --- | --- | --- | --- | --- | --- | --- | --- | --- | --- | --- | --- | --- | --- | --- | --- | --- | --- | --- | --- | --- | --- | --- | --- | --- | --- | --- | --- | --- | --- | --- | --- | --- | --- | --- | --- | --- | --- | --- | --- | --- | --- | --- | --- | --- | --- | --- | --- | --- | --- | --- | --- | --- | --- | --- | --- | --- | --- | --- | --- | --- | --- | --- | --- | --- | --- | --- | --- | --- | --- | --- | --- | --- | --- | --- | --- | --- | --- | --- | --- | --- | --- | --- | --- | --- | --- | --- | --- | --- | --- | --- | --- | --- | --- | --- | --- | --- | --- | --- | --- | --- | --- | --- | --- | --- | --- | --- | --- | --- | --- | --- | --- | --- | --- | --- | --- | --- | --- | --- | --- | --- | --- | --- | --- | --- | --- | --- | --- | --- | --- | --- | --- | --- | --- | --- | --- | --- | --- | --- | --- | --- | --- | --- | --- | --- | --- | --- | --- | --- | --- | --- | --- | --- | --- | --- | --- | --- | --- | --- | --- | --- | --- | --- | --- | --- | --- | --- | --- | --- | --- | --- | --- | --- | --- | --- | --- | --- | --- | --- | --- | --- | --- | --- | --- | --- | --- | --- | --- | --- | --- | --- | --- | --- | --- | --- | --- | --- | --- | --- | --- | --- | --- | --- | --- | --- | --- | --- | --- | --- | --- | --- | --- | --- | --- | --- | --- | --- | --- | --- | --- | --- | --- | --- | --- | --- | --- | --- | --- | --- | --- | --- | --- | --- | --- | --- | --- | --- | --- | --- | --- | --- | --- | --- | --- | --- | --- | --- | --- | --- | --- | --- | --- | --- | --- | --- | --- | --- | --- | --- | --- | --- | --- | --- | --- | --- | --- | --- | --- | --- | --- | --- | --- | --- | --- | --- | --- | --- | --- | --- | --- | --- | --- | --- | --- | --- | --- | --- | --- | --- | --- | --- | --- | --- | --- | --- | --- | --- | --- | --- | --- | --- | --- | --- | --- | --- | --- | --- | --- | --- | --- | --- | --- | --- | --- | --- | --- | --- | --- | --- | --- | --- | --- | --- | --- | --- | --- | --- | --- | --- | --- | --- | --- | --- | --- | --- | --- | --- | --- | --- | --- | --- | --- | --- | --- | --- | --- | --- | --- | --- | --- | --- | --- | --- | --- | --- | --- | --- | --- | --- | --- | --- | --- | --- | --- | --- | --- | --- | --- | --- | --- | --- | --- | --- | --- | --- | --- | --- | --- | --- | --- | --- | --- | --- | --- | --- | --- | --- | --- | --- | --- | --- | --- | --- | --- | --- | --- | --- | --- | --- | --- | --- | --- | --- | --- | --- | --- | --- | --- | --- | --- | --- | --- | --- | --- | --- | --- | --- | --- | --- | --- | --- | --- | --- | --- | --- | --- | --- | --- | --- | --- | --- | --- | --- | --- | --- | --- | --- | --- | --- | --- | --- | --- | --- | --- | --- | --- | --- | --- | --- | --- | --- | --- | --- | --- | --- | --- | --- | --- | --- | --- | --- | --- | --- | --- | --- | --- | --- | --- | --- | --- | --- | --- | --- | --- | --- | --- | --- | --- | --- | --- | --- | --- | --- | --- | --- | --- | --- | --- | --- | --- | --- | --- | --- | --- | --- | --- | --- | --- | --- | --- | --- | --- | --- | --- | --- | --- | --- | --- | --- | --- | --- | --- | --- | --- | --- | --- | --- | --- | --- | --- | --- | --- | --- | --- | --- | --- | --- | --- | --- | --- | --- | --- | --- | --- | --- | --- | --- | --- | --- | --- | --- | --- | --- | --- | --- | --- | --- | --- | --- | --- | --- | --- | --- | --- | --- | --- | --- | --- | --- | --- | --- | --- | --- | --- | --- | --- | --- | --- | --- | --- | --- | --- | --- | --- | --- | --- | --- | --- | --- | --- | --- | --- | --- | --- | --- | --- | --- | --- | --- | --- | --- | --- | --- | --- | --- | --- | --- | --- | --- | --- | --- | --- | --- | --- | --- | --- | --- | --- | --- | --- | --- | --- | --- | --- | --- | --- | --- | --- | --- | --- | --- | --- | --- | --- | --- | --- | --- | --- | --- | --- | --- | --- | --- | --- | --- | --- | --- | --- | --- | --- | --- | --- | --- | --- | --- | --- | --- | --- | --- | --- | --- | --- | --- | --- | --- | --- | --- | --- | --- | --- | --- | --- | --- | --- | --- | --- | --- | --- | --- | --- | --- | --- | --- | --- | --- | --- | --- | --- | --- | --- | --- | --- | --- | --- | --- | --- | --- | --- | --- | --- | --- | --- | --- | --- | --- | --- | --- | --- | --- | --- | --- | --- | --- | --- | --- | --- | --- | --- | --- | --- | --- | --- | --- | --- | --- | --- | --- | --- | --- | --- | --- | --- | --- | --- | --- | --- | --- | --- | --- | --- | --- | --- | --- | --- | --- | --- | --- | --- | --- | --- | --- | --- | --- | --- | --- | --- | --- | --- | --- | --- | --- | --- | --- | --- | --- | --- | --- | --- | --- | --- | --- | --- | --- | --- | --- | --- | --- | --- | --- | --- | --- | --- | --- | --- | --- | --- | --- | --- | --- | --- | --- | --- | --- | --- | --- | --- | --- | --- | --- | --- | --- | --- | --- | --- | --- | --- | --- | --- | --- | --- | --- | --- | --- | --- | --- | --- | --- | --- | --- | --- | --- | --- | --- | --- | --- | --- | --- | --- | --- | --- | --- | --- | --- | --- | --- | --- | --- | --- | --- | --- | --- | --- | --- | --- | --- | --- | --- | --- | --- | --- | --- | --- | --- | --- | --- | --- | --- | --- | --- | --- | --- | --- | --- | --- | --- | --- | --- | --- | --- | --- | --- | --- | --- | --- | --- | --- | --- | --- | --- | --- | --- | --- | --- | --- | --- | --- | --- | --- | --- | --- | --- | --- | --- | --- | --- | --- | --- | --- | --- | --- | --- | --- | --- | --- | --- | --- | --- | --- | --- | --- | --- | --- | --- | --- | --- | --- | --- | --- | --- | --- | --- | --- | --- | --- | --- | --- | --- | --- | --- | --- | --- | --- | --- | --- | --- | --- | --- | --- | --- | --- | --- | --- | --- | --- | --- | --- | --- | --- | --- | --- | --- | --- | --- | --- | --- | --- | --- | --- | --- | --- | --- | --- | --- | --- | --- | --- | --- | --- | --- | --- | --- | --- | --- | --- | --- | --- | --- | --- | --- | --- | --- | --- | --- | --- | --- | --- | --- | --- | --- | --- | --- | --- | --- | --- | --- | --- | --- | --- | --- | --- | --- | --- | --- | --- | --- | --- | --- | --- | --- | --- | --- | --- | --- | --- | --- | --- | --- | --- | --- | --- | --- | --- | --- | --- | --- | --- | --- | --- | --- | --- | --- | --- | --- | --- | --- | --- | --- | --- | --- | --- | --- | --- | --- | --- | --- | --- | --- | --- | --- | --- | --- | --- | --- | --- | --- | --- | --- | --- | --- | --- | --- | --- | --- | --- | --- | --- | --- | --- | --- | --- | --- | --- | --- | --- | --- | --- | --- | --- | --- | --- | --- | --- | --- | --- | --- | --- | --- | --- | --- | --- | --- | --- | --- | --- | --- | --- | --- | --- | --- | --- | --- | --- | --- | --- | --- | --- | --- | --- | --- | --- | --- | --- | --- | --- | --- | --- | --- | --- | --- | --- | --- | --- | --- | --- | --- | --- | --- | --- | --- | --- | --- | --- | --- | --- | --- | --- | --- | --- | --- | --- | --- | --- | --- | --- | --- | --- | --- | --- | --- | --- | --- | --- | --- | --- | --- | --- | --- | --- | --- | --- | --- | --- | --- | --- | --- | --- | --- | --- | --- | --- | --- | --- | --- | --- | --- | --- | --- | --- | --- | --- | --- | --- | --- | --- | --- | --- | --- | --- | --- | --- | --- | --- | --- | --- | --- | --- | --- | --- | --- | --- | --- | --- | --- | --- | --- | --- | --- | --- | --- | --- | --- | --- | --- | --- | --- | --- | --- | --- | --- | --- | --- | --- | --- | --- | --- | --- | --- | --- | --- | --- | --- | --- | --- | --- | --- | --- | --- | --- | --- | --- | --- | --- | --- | --- | --- | --- | --- | --- | --- | --- | --- | --- | --- | --- | --- | --- | --- |
|  |  |  |  |  |  |  |  |  |  |  |  |  |  |  |  |  |  |  |  |
|  |  |  |  |  |  |  |  |  |  |  |  |  |  |  |  |  |  |  |  |
|  |  |  |  |  |  |  |  |  |  |  |  |  |  |  |  |  |  |  |  |
|  |  |  |  |  |  |  |  |  |  |  |  |  |  |  |  |  |  |  |  |
|  |  |  |  |  |  |  |  |  |  |  |  |  |  |  |  |  |  |  |  |
|  |  |  |  |  |  |  |  |  |  |  |  |  |  |  |  |  |  |  |  |
|  |  |  |  |  |  |  |  |  |  |  |  |  |  |  |  |  |  |  |  |
|  |  |  |  |  |  |  |  |  |  |  |  |  |  |  |  |  |  |  |  |
|  |  |  |  |  |  |  |  |  |  |  |  |  |  |  |  |  |  |  |  |
|  |  |  |  |  |  |  |  |  |  |  |  |  |  |  |  |  |  |  |  |
|  |  |  |  |  |  |  |  |  |  |  |  |  |  |  |  |  |  |  |  |
|  |  |  |  |  |  |  |  |  |  |  |  |  |  |  |  |  |  |  |  |
|  |  |  |  |  |  |  |  |  |  |  |  |  |  |  |  |  |  |  |  |
|  |  |  |  |  |  |  |  |  |  |  |  |  |  |  |  |  |  |  |  |
|  |  |  |  |  |  |  |  |  |  |  |  |  |  |  |  |  |  |  |  |
|  |  |  |  |  |  |  |  |  |  |  |  |  |  |  |  |  |  |  |  |
|  |  |  |  |  |  |  |  |  |  |  |  |  |  |  |  |  |  |  |  |
|  |  |  |  |  |  |  |  |  |  |  |  |  |  |  |  |  |  |  |  |
|  |  |  |  |  |  |  |  |  |  |  |  |  |  |  |  |  |  |  |  |
