# Supplementary material for: Potential risk of Batrachochytrium salamandrivorans in Mexico
Source: PLoS One. 2019 Feb 12;14(2):e0211960. doi: 10.1371/journal.pone.0211960 (PMC6372179; doi:10.1371/journal.pone.0211960)
Supplement: S7 Table — (DOCX) [file pone.0211960.s011.docx]

| Hotspot identification | Hotspot area | Species |
| --- | --- | --- |
| 1 | TVB | *Ambystoma granulosum, Ambystoma rivulare, Ambystoma velasci, Chiropterotriton orculus, Isthmura bellii, Pseudoeurycea leprosa, Pseudoeurycea robertsi* |
| 2 | TVB | *Ambystoma granulosum, Ambystoma rivulare, Ambystoma velasci, Isthmura bellii, Pseudoeurycea leprosa* |
| 3 | TVB | *Ambystoma altamirani, Ambystoma rivulare, Aquiloerycea cephalica, Chiropterotriton orculus, Isthmura bellii, Pseudoeurycea leprosa, Pseudoeurycea tlilicxitl* |
| 4 | TVB | *Ambystoma velasci, Aquiloerycea cephalica, Chiropterotriton chico, Chiropterotriton dimidiatus, Pseudoeurycea leprosa* |
| 5 | TVB | *Ambystoma velasci, Aquiloerycea cephalica, Chiropterotriton orculus, Isthmura bellii, Pseudoeurycea leprosa* |
| 6 | TVB | *Ambystoma leorae, Ambystoma velasci, Aquiloerycea cephalica, Chiropterotriton orculus, Isthmura bellii, Pseudoeurycea leprosa, Pseudoeurycea tlilicxitl* |
| 7 | TVB | *Aquiloerycea cephalica, Pseudoeurycea gadovii, Pseudoeurycea leprosa, Pseudoeurycea lynchi, Pseudoeurycea melanomolga* |
| 8 | TVB | *Aquiloerycea cephalica, Aquiloeurycea cafetalera, Chiropterotriton sp. E, Chiropterotriton lavae, Isthmura gigantea, Pseudoeurycea leprosa, Pseudoeurycea lynchi, Thorius minydemus, Thorius munificus* |
| 9 | Los Tuxtlas | *Bolitoglossa alberchi, Bolitoglossa platydactyla, Bolitoglossa rufescens, Pseudoeurycea orchimelas, Pseudoeurycea werleri, Thorius narismagnus* |
| 10 | Los Tuxtlas | *Bolitoglossa alberchi, Bolitoglossa platydactyla, Bolitoglossa rufescens, Pseudoeurycea orchimelas, Pseudoeurycea werleri* |
| 11 | SMS Guerrero | *Pseudoeurycea ahuitzotl, Pseudoeurycea tenchalli, Pseudoeurycea teotepec, Pseudoeurycea tlahcuiloh, Thorius grandis* |
| 12 | Northern Oaxaca | *Chiropterotriton sp. K, Isthmura boneti, Pseudoeurycea smithi, Pseudoeurycea unguidentis, Thorius narisovalis, Thorius pulmonaris* |
| 13 | SMS Chiapas | *Bolitoglossa engelhardti, Bolitoglossa flavimembris, Bolitoglossa franklini, Bolitoglossa occidentalis, Bolitoglossa rostrata, Pseudoeurycea brunnata, Pseudoeurycea goebeli, Pseudoeurycea rex* |
